# Multi-drug tolerance in *Leishmania* persister-like cells

**DOI:** 10.1038/s42003-026-10456-w

**Published:** 2026-06-12

**Authors:** Allison Aroni-Soto, Pieter Monsieurs, Odin Goovaerts, Kaoutar Choukri, Basudha Khanal, Wim Adriaensen, Jean-Claude Dujardin, Michael P. Barrett, Malgorzata A. Domagalska

**Affiliations:** 1 Institute of Tropical Medicine, Experimental Parasitology Unit, Antwerp, Belgium; 2 Department of Biomedical Sciences, University of Antwerp, Antwerp, Belgium; 3 Institute of Tropical Medicine, Molecular Parasitology Unit, Antwerp, Belgium; 4 Institute of Tropical Medicine, Clinical Immunology Unit, Antwerp, Belgium; 5 Department of Microbiology, BP Koirala Institute of Health Sciences, Dharan, Nepal; 6 School of Infection & Immunity, College of Medical, Veterinary and Life Sciences, University of Glasgow, Glasgow, UK

**Keywords:** Parasite biology, Antimicrobial resistance

## Abstract

Leishmaniasis is caused by parasitic protozoa of the genus *Leishmania* and is found widely across the tropics and sub-tropics, afflicting hundreds of thousands of people. The disease is notoriously difficult to treat. Here, we present evidence of the existence of persister-like cells in cultured *Leishmania* populations, induced upon exposure to normally lethal doses of antimony, a still used anti-leishmanial. Persisters are a small fraction of non-proliferative cells with reduced metabolism that are adapted to withstand a variety of environmental assaults, including lethal doses of antimicrobials. We show that *Leishmania* persister-like cells survive lethal doses of antimonials by adopting a quiescence phenotype characterised by reduced proliferation, constrained metabolism, diminished mitochondrial membrane potential and downregulated transcription of core metabolic pathways. What is more, these cells demonstrate cross-tolerance to other anti-leishmanial drugs. Wild-type persister-like cells reverted to similar levels of drug susceptibility once the antimony-induced pressure was removed. Surprisingly, cells from strains with genetic changes causing resistance to antimony acquired a level of hyper-resistance after transient passage through the quiescent state, without further genetic change. Our results demonstrate the extreme versatility of this eukaryotic pathogen in adaptation to drug pressure and highlight the need for the development of new anti-leishmanials targeting non-proliferative forms.

## Abstract (web summary)

*Leishmania* persister-like cells survive lethal doses of antimonials by adopting a quiescent state, they demonstrate cross-tolerance to other anti-leishmanial drugs and become hyper-resistant in the presence of pre-existing drug resistance.

## Introduction

The ability of microorganisms to resist the killing action of antimicrobial agents is emerging as one of the most serious public health threats and has been observed for antivirals, antibiotics, antifungals, and also antiparasitic compounds^1^. Understanding the mechanisms that allow pathogens to survive chemotherapy is essential. Several molecular mechanisms can underlie this phenomenon, but the most extensively studied one is drug resistance caused by acquired, heritable genetic changes, usually point mutations or insertion/deletion within genes in which these mutations enable cells to prevent the drug’s toxic capacity^2,3^. Importantly, resistant pathogens remain metabolically active and can grow and replicate in the presence of drugs. However, microorganisms can also adopt a physiological state of transient quiescence, manifested by non-proliferation and reduced metabolism. This leads to non-heritable drug tolerance in cases where drug activity depends upon metabolic activities suppressed in quiescence^4^. This can affect whole-microbial populations, but heterogeneity within microbial populations may allow the survival of a small number of specialized quiescent cells, termed ‘persisters’, in the presence of an antimicrobial agent^5^. After the removal of drug pressure, persisters resume growth and switch back to their drug-sensitive phenotype. The presence of persisters is of particular importance as their quiescent phenotype, associated with exposure to a single drug, often leads to multidrug tolerance^6–8^.

Quiescence is a non-proliferative, physiological resting state that can be adopted by many infectious microorganisms, including bacteria, fungi, and some protozoan parasites as a strategy to survive nutrient limitation, host immunity, and chemotherapy^9^. This reversible state is characterized by an active and regulated cellular and molecular remodelling programme resulting in reduced metabolism, transcription, and translation^9,10^. Importantly, while overall metabolism is switched into an energy-saving mode, crucial processes allowing the survival of the cells under stress conditions remain active, and some processes are even selectively upregulated in the quiescent state^11^.

*Leishmania* are digenetic protozoan parasites that cycle between the sand fly vector and mammalian host. They cause a spectrum of diseases, the most severe form being visceral leishmaniasis (VL), which is lethal if left untreated. *Leishmania donovani* is the etiological agent of VL in the Indian subcontinent (ISC) and in East Africa. Antimonials (Sb) were the first drugs used in the treatment of VL, but they have been replaced first by miltefosine and later by Amphotericin B (AmB) in the ISC due to loss of efficacy attributed to the emergence of drug resistance^12–15^.

In terms of drug responses in *Leishmania*, most research has focused on identification of potential drug resistance signatures in natural populations and in experimental evolution studies^16–20^. However, recent evidence points to the existence of quiescence and its role in adaptation to drug pressure^21–25^. Quiescence in *Leishmania* was inferred in mice when amastigotes of *L. mexicana* were shown to have a very low replication rate, together with low rates of RNA synthesis, protein turnover, and membrane lipid synthesis^26^. Another mouse study revealed heterogeneity among *L. major* amastigotes with non-replicating parasites distinguished from slowly replicating populations^27^. Another study of *L. mexicana* in mice revealed quiescent populations within collagen-rich mesothelium surrounding lesions, where miltefosine accumulation was also limited^24^. Using an in vitro antimony-exposure model, we showed that *L. lainsoni* parasites exhibit hallmarks of a quiescence state and that drug-tolerant and persister-like cells of genetically diverse strains of *L. braziliensis* could be detected under high doses of potassium antimonium tartrate (PAT)^22,23^. Recently, Dirkx and colleagues showed that long-term hematopoietic stem cells provide a parasite niche during treatment failure in an animal model of VL infection^28^. Bone marrow stem cell-associated parasites were proposed to enter into quiescence after 2–3 replication cycles with parasites emerging post-quiescence with increased apparent infectivity^25^.

In this study, we addressed: (i) the role of quiescence and population heterogeneity in the survival of *L. donovani* promastigotes under normally lethal doses of PAT, and (ii) whether the presence of genomically defined resistance to antimony has an impact on the quiescence and persister phenotypes under PAT exposure.

## Results

### Isogenic *L. donovani* lines exposed to normally lethal doses of PAT exhibit intrinsic population heterogeneity in their drug susceptibility

To address whether heterogeneity within populations and quiescence plays a role in response to drug pressure in *L. donovani*, we developed an experimental model, in which we focused on the detection of a small subpopulation of surviving persister-like cells. Specifically, we aimed to identify cells that i) constituted a small subpopulation within isogenic lines, ii) could survive in the presence of a normally lethal dose of a drug and iii) showed a reduction in growth and metabolism associated with a quiescent state when compared with the majority of cells. We chose trivalent antimony (PAT), as we showed previously that quiescent cells could be observed in the presence of high doses of this drug in a dermotropic species of *Leishmania*, *L. braziliensis*^22^.

We selected eleven cloned, genomically characterized strains of *L. donovani*^16^, with different levels of susceptibility to antimony. Hereby, we aimed to study the potential interplay between different heritable, genetically defined forms of drug resistance and quiescence in parasite survival under PAT pressure. We generated rEGFP expressing lines for each of these strains to monitor transcriptional activity of the rDNA locus as a negative biomarker of quiescence during exposure to PAT^29^.

**Fig. 1:**
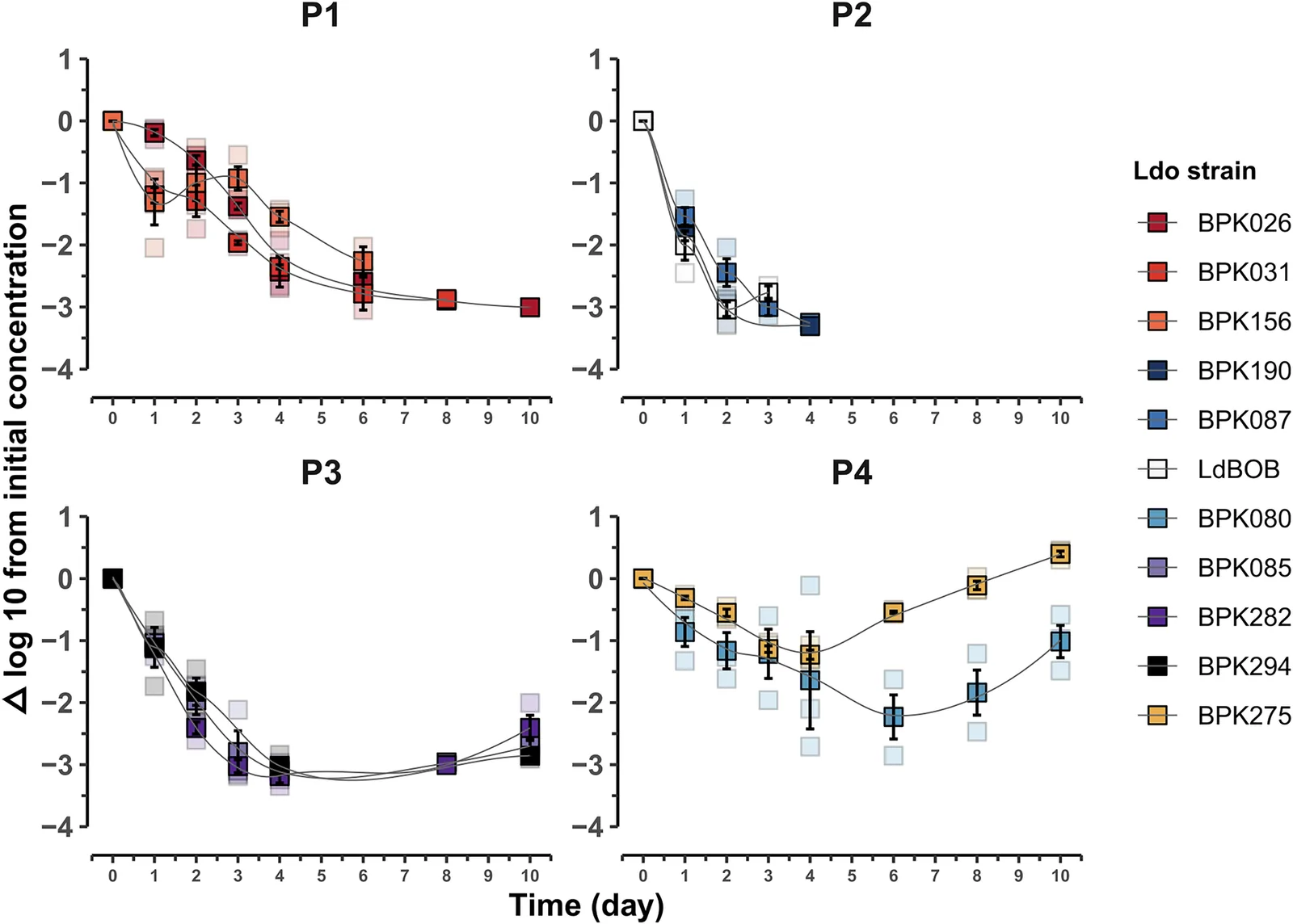
Patterns of survival of *L. donovani* strains under normally lethal PAT concentrations. Time-kill curves under prolonged PAT exposure (see separate strain-specific curves in Supplementary Fig. 3) showed four different viability profiles (Profile 1–4). Data represents the reduction in promastigote concentration relative to the initial inoculum, the viable parasites were detected by flow cytometry. The results reflect the mean ± SEM of three biological replicates and the individual data points are represented by semi-transparent symbols in the respective strain color.

In the first step, we exposed promastigotes of each line to a range of PAT concentrations to determine the lowest concentration (Supplementary Fig. 1 and Supplementary Table 1) at which no metabolic activity could be detected in a resazurin based assay measured at the level of the whole population^30^. By identifying normally lethal doses of PAT specific for each strain, we aimed to establish comparable experimental conditions in which physiologically quiescent-like cells would be observed for each strain, as shown for bacteria^4,30^. We then subjected each line to the respective strain-specific normally lethal PAT doses over 10 days (with PAT application repeated at day 5), and we monitored viability and growth (Supplementary Table 2 and Supplementary Fig. 2) and established the time-kill curves (Fig. 1 and Supplementary Fig. 3) during the exposure. Overall, we identified four broad viability profiles in response to PAT pressure (Fig. 1 and Supplementary Table 2).

Profile 1 (P1) observed in the ISC1 parasites was characterized by continuous reduction of viability, with the majority of the parasites killed in the first 6 days, while a small remaining population was killed at a slower rate over the last 4 days, allowing those cells to persist. In profile 2 (P2), we observed rapid killing of cells in the first 2 days, which resulted in no viable parasites by day 8. Profile 3 (P3) could be distinguished by fast reduction in viability over the first 3–4 days, followed by a slower killing rate until days 6–8. Surprisingly, in the last 2 days of exposure to PAT, a clear increase in the number of viable parasites was observed. Finally, the last profile (profile 4, P4) is defined by mild reduction in viability, followed by a short period of no visible growth, then a subsequent increase in the number of viable cells (Fig. 1 and Supplementary Table 2).

### *L. donovani* lines adopt a quiescent state to survive PAT pressure

**Fig. 2:**
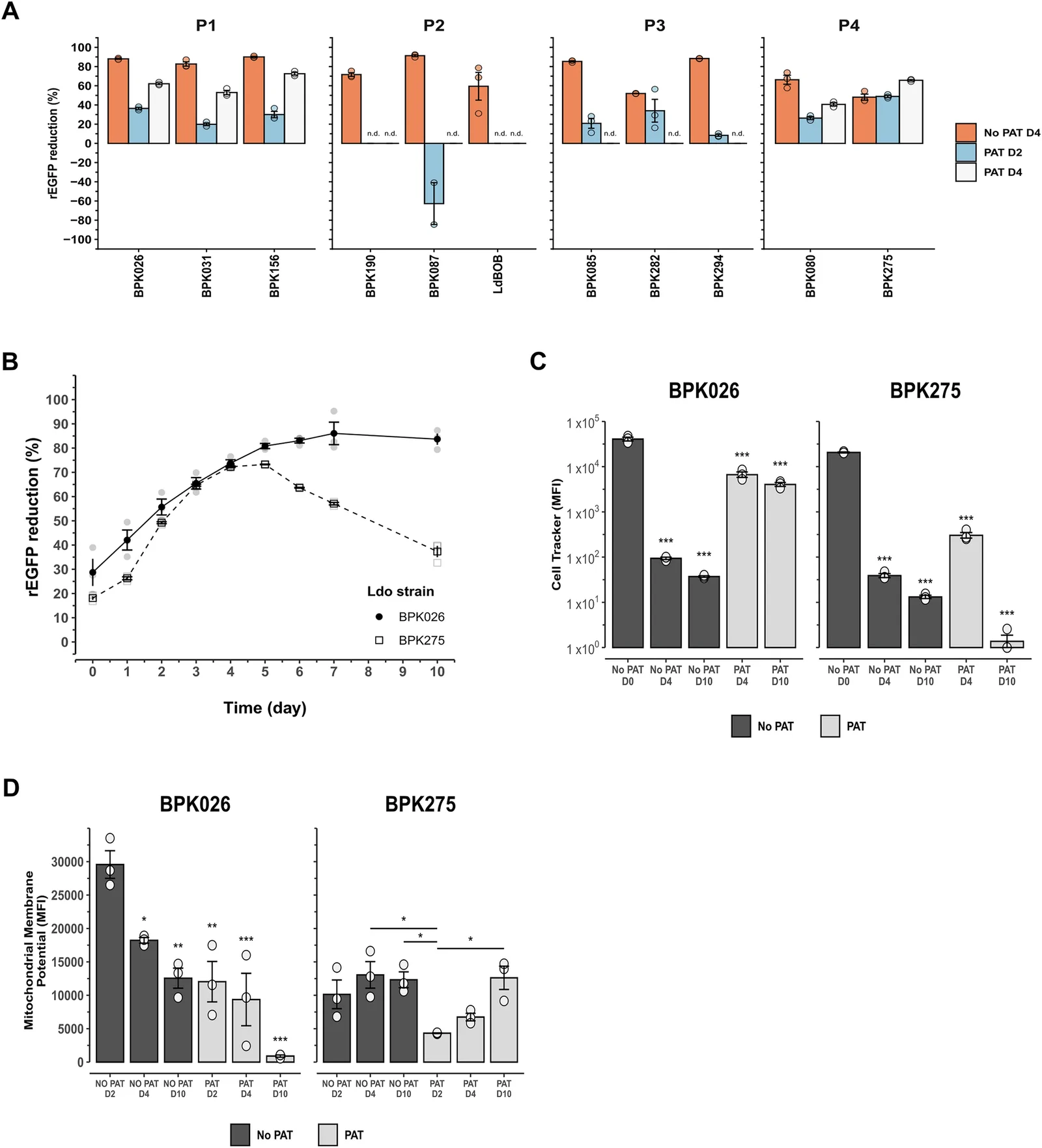
Quiescence features of *L. donovani* promastigotes under prolonged PAT exposure. **A** rEGFP expression reduction in percentage in P1-P4 profiles under normally lethal doses of PAT exposure for 2 (PAT D2) and 4 (PAT D4) days. A group without exposure for 4 days was included (No PAT D4). In certain samples, rEGFP expression data are not reported due to a limited number of viable cells, indicated as ‘n.d.’ (not determined). **B** rEGFP expression reduction in percentage in BPK026 and BPK275 from day 0 to 10. **C** Proliferation status measured by CellTracker in promastigotes from BPK026 and BPK275 under PAT exposure (PAT) with the respective no-drug controls (No PAT). The results were compared to the initial staining point of the parasites (No PAT D0). Each round of replication dilutes the CellTracker dye resulting in decreased fluorescence signal, thus fluorescence levels are expected to remain unchanged in non-proliferating cells. In this assay, logarithmic promastigotes were labelled by CellTracker (D0) and either exposed to PAT at a normally lethal dose (PAT) or grown in the control medium (No PAT), and the fluorescence signal was evaluated during short- (D4) and long-term (D10) PAT exposure. **D** Measurement of the mitochondrial membrane potential with MitoProbe ™ DilC by flow cytometry in promastigotes exposed for 2, 4, and 10 days to strain-specific PAT normally lethal dose (PAT); and the respective controls without drug (No PAT). The results were compared to promastigotes at the logarithmic state (No PAT D2). DilC is a permeable cationic dye that accumulates across the mitochondrial membrane when the interior of the mitochondrion is negatively charged compared to its outside. **A**, **B** The rEGFP expression reduction in percentage was calculated by comparison of the signal of each group to promastigotes without PAT exposure at the logarithmic state. The data reflects the mean ± SEM of three biological replicates, with the corresponding individual data points shown as semi-transparent symbols (**A**, **B**) or as circles (**C**, **D**). The asterisks represent the statistically significant difference after Tukey’s post hoc test; *P < 0.05, **P < 0.01, ***P < 0.001.

Given the initial drug-related reduction in growth in all tested lines, we addressed whether the PAT-exposed cells adopt a quiescent state. We addressed this first by analyzing the reduction of rEGFP expression -a proxy of metabolic activity- in all of the lines exposed to PAT at day 2, and 4 (PAT D2 and PAT D4, respectively), as compared to proliferative promastigotes. We included, as a positive control, stationary promastigotes (No PAT D4), which are known to have properties of quiescent cells^23^. In all lines of P1/P4 we observed a progressive reduction of rEGFP under PAT pressure, reaching comparable or slightly lower levels to those observed in No PAT D4 (Fig. 2A). In parasites belonging to P2 and P3, measurements were possible only at D2 (all lines in P3 and only BPK087 in P2) due to rapid killing in the lines of these profiles, which resulted in an insufficient number of viable cells for reliable quantification of rEGFP expression. As in other lines, we detected reduction in rEGFP expression at D2 in all three P3 lines. Surprisingly, we observed an upregulation of rEGFP in BPK087, suggesting an inability to enter quiescence under PAT pressure (62.7% of increase, Fig. 2A). As expected, all lines that were able to survive drug pressure resumed normal growth after transfer to standard growth conditions without PAT (Supplementary Table 3).

To confirm the quiescent status of the viable cells under PAT, we selected two lines, BPK026 and BPK275, which present the most extreme viability profiles (P1 and P4, respectively) and extreme genomic signatures of susceptibility to PAT (no amplification of H locus and wild type *AQP1* for BPK026 and amplification of H locus and truncated AQP1 for BPK275) for more in-depth characterization. First, we measured rEGFP expression at 24 h intervals until day 7, and at the last day of PAT exposure. Both lines showed an overall rEGFP decrease among viable cells over the first 4 days of PAT pressure (Fig. 2B), matching the observed growth arrest (Supplementary Figure 4A). This decrease continued for BPK026 over the next 6 days, reaching a reduction in expression value of 83.1%. In contrast, rEGFP expression for BPK275 remained stable until day 5, followed by a clear increase by day 10 (Fig. 2B). This is consistent with the apparent reactivation and increase in the percentage of viable and proliferative cells towards the end of drug exposure (Supplementary Fig. 4B). Second, we assessed whether parasite replication is arrested/reduced using CellTracker (tracking cell proliferation) combined with SYTOX Blue dye (viability, Fig. 2C). In both lines, No PAT parasites showed clear proliferation as indicated by a decrease in fluorescence, reaching 1.97–1.57 to 1.59–1.12 log_10_ values for BPK026 and BPK275, respectively (Fig. 2C). PAT-exposed parasites by contrast showed less dilution (Fig. 2C). In BPK026, the CellTracker signal decreased slightly from 4.61 to 3.82 log_10_ values at PAT D4, and remained almost the same at PAT D10 (3.61 log_10_ values) indicating an overall low/no proliferation of PAT-exposed cells. In the case of BPK275, the signal in PAT D4 (2.48 log_10_ values) decreased as compared to D0 (4.31 log_10_ values), but it was still higher than fluorescence in the No PAT D4 parasites (1.59 log_10_ values). No CellTracker signal was detected in PAT D10; this indicates that during the early exposure to PAT, BPK275 parasites transiently reduce their proliferation, while resuming active proliferation later.

Next, we investigated the mitochondrial membrane potential as an indicator of mitochondrial activity, which is known to be reduced in quiescent *Leishmania* cells^23^. In the absence of PAT, BPK026 showed high potential in logarithmic promastigotes, and this decreased significantly in the stationary phase. Unexpectedly, in BPK275, the mitochondrial membrane potential was much lower in logarithmic parasites in comparison to BPK026 (29,569 ± 2071 and 10,142 ± 2147 MFI values, in BPK026 and BPK275, respectively, Fig. 2D). Under PAT pressure, patterns were also markedly different between lines. In BPK026, early PAT-exposed promastigotes showed a similar potential to late stationary (D10) promastigotes, which further decreased to 899 ± 165 MFI values at day 10 (Fig. 2D). In the PAT-exposed BPK275, the mitochondrial potential was the lowest at D2 (4326 ± 47 MFI value), and it gradually increased, reaching 12,611 ± 1741 MFI value at D10 (Fig. 2D).

Thus, based on the rEGFP, CellTracker, and mitochondrial potential measurements, we concluded that both lines exhibited features of quiescence during their exposure to normally lethal doses of PAT. Though in BPK275, the line with genetic resistance to PAT, the period of quiescence was transient, and the quiescence state appeared to be less deep.

### PAT resistant and sensitive *L. donovani* strains exhibit common molecular signatures of quiescence

**Fig. 3:**
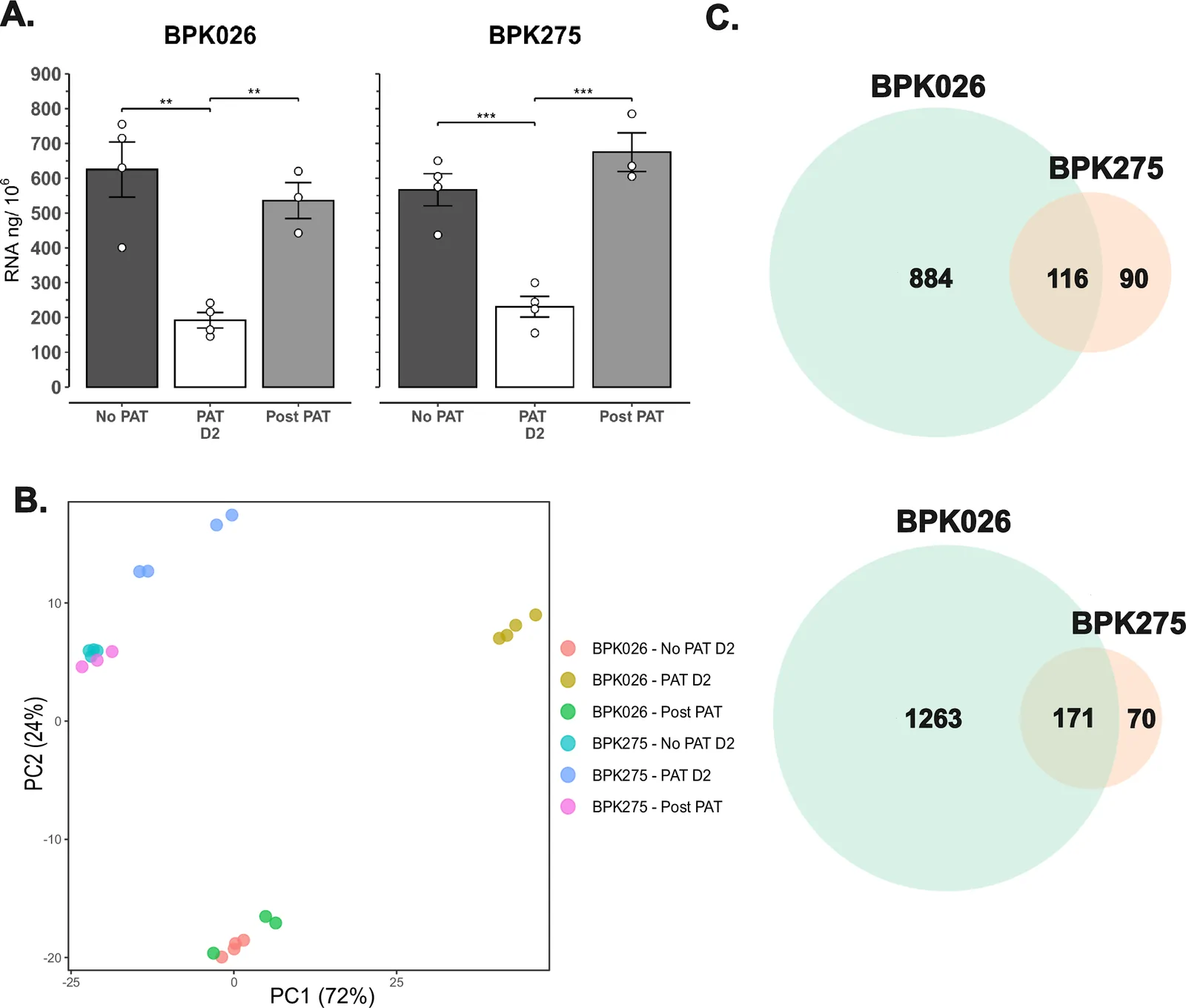
Total RNA measurements and transcriptomic adaptations in quiescent *L. donovani* promastigotes. **A** RNA quantity of *L. donovani* promastigotes under PAT exposure for 2 days (PAT D2), logarithmic parasites recovered after PAT exposure (Post PAT), and controls without exposure at the logarithmic phase (No PAT) per million cells. The figure shows the mean of three/four biological replicates ± SEM, and the asterisks represent the statistically significant difference after Tukey’s post-hoc test: *P < 0.05, **P < 0.01, ***P < 0.001. **B** PCA was performed on variance-stabilized expression values (VST, DESeq2) to visualize global expression patterns across samples. Each point represents one biological replicate and is colored according to experimental condition. The percentages on the axes indicate the proportion of total variance explained by Principle Component 1 and Principle Component 2. **C** Venn diagram showing the number of significantly downregulated (upper diagram) and upregulated (lower diagram) genes in BPK026 and BPK275 at D2 following PAT treatment compared to controls at D2 without PAT treatment (No PAT). Genes were considered significantly up- or downregulated if the absolute log2 fold change was higher than 1 and the false discovery rate (FDR) was < 0.05.

To further characterize molecular adaptations of these quiescent parasites, we performed total RNA measurements and transcriptomic analyses of BPK026 and BPK275 lines. Reduction in total RNA content was previously associated with quiescent state in several eukaryotic organisms^31–33^, including another *Leishmania* species, *Leishmania lainsoni*^23^. To address this, we collected and extracted RNA from logarithmic-phase parasites before PAT exposure (No PAT D2), recovered from drug pressure (Post PAT) and quiescent cells exposed to PAT for 2 days (PAT D2). We observed approximately 2-3x reduction in total RNA content/10^6^ cells in both strains at PAT D2 (191.9 ± 22.3 and 230.8 ± 29.8 values, respectively), in comparison to replicative No PAT D2 and Post-PAT promastigotes, which both showed comparable values (625.1 ± 79.3, and 535.8 ± 51.4 for BPK026, 566.8 ± 45.9, and 675.0 ± 55.7 for BPK275 (Fig. 3A). Next, we performed transcriptomic analysis to identify transcripts and molecular processes modulated in these quiescent parasites. The sequencing statistics, mapping statistics, count tables can be found in Supplementary Data 1. PCA (PC1 = 72%, PC2 = 24% of the variance explained) showed that (i) there was clear separation between the strains based on genetic background, (ii) the drug-exposed parasites cluster together, and away from the non-exposed cells, with the samples of BPK026 being further apart than the ones of BPK275, (iii) both BPK026 and BPK275 no PAT and Post PAT samples clustered together (Fig. 3B). Next, using DESeq2 package we performed several pairwise differential expression analyses (summarized in Supplementary Table 4 and Supplementary Data 2–4). Here we highlight the key findings.

First, we focused on identifying molecular signatures of quiescent *Leishmania* cells. We found that the PAT-exposed (quiescent) antimony sensitive BPK026 line exhibited a higher number of differentially expressed genes than the drug resistant BPK275 (total of 2592 and 447, respectively (Supplementary Table 4). Compared to No PAT promastigotes, PAT-exposed cells showed 1592 and 241 transcripts with reduced abundance, and 1000 and 206 with increased levels in BPK026 and BPK275, respectively. To discover which molecular pathways are modulated in these quiescent cells, we performed KEGG enrichment analysis (Supplementary Data 5). In this analysis, pathway enrichment was assessed per condition using differentially expressed genes and a hypergeometric model, with only significantly enriched pathways (adjusted *p* < 0.05) retained. In PAT exposed BPK026, we found almost all core metabolic pathways related to carbohydrate, energy, nucleotide, and amino acid metabolism, as well as several lipid metabolism-related pathways to be downregulated (Supplementary Data 5). The overall reduction in cellular activity was further evidenced by downregulation of cytoskeleton related proteins, transporters, chromosome-associated proteins, signalling components, chaperones and folding catalysts and processes related to translation, exosome and nucleocytoplasmic transport (RNA transport). In contrast, only few pathways were enriched in the quiescent cell population of BPK026. These included Glycosylphosphatidylinositol (GPI)-anchor biosynthesis, ribosome and ribosome biogenesis, autophagy, mitophagy and lipoic acid metabolism (Supplementary Data 5). In PAT-exposed BPK275, a lower number of downregulated pathways could be identified, but we observed a similar trend as in BPK026 with decreases in core metabolic pathways, cytoskeleton related proteins, transporters, chromosome-associated proteins, signalling components, chaperones and folding catalysts and processes related to exosome and nucleocytoplasmic transport (RNA transport) (Supplementary Data 5). Analysis of upregulated pathways in quiescent BPK275 revealed enrichment in genes related to autophagy and mitophagy, membrane trafficking, and membrane transporters.

The trend of downregulation of core cellular processes was confirmed when we investigated which metabolic pathways would be enriched in DE-expressed genes in PAT-exposed cells, this time common for both strains (Supplementary Data 5). Transcripts related to autophagy (e.g.,* ATG9*) and membrane trafficking showed higher abundance in PAT-exposed cells of both strains. In addition, transcripts encoding selected amastins, amastin-like proteins and amastin surfaceglycoproteins, putative kinesin k39, tryparedoxin peroxidase, glycosomal phosphoenolpyruvate carboxykinase and calcineurin B subunit, and several so far uncharacterized hypothetical proteins were also upregulated in both strains (Supplementary Table 4). Collectively, these transcriptomic results confirm strong metabolic downregulation accompanied by transcriptional reprogramming in both strains exposed to PAT, which is consistent with the adoption of a quiescent state. Accordingly, this transcriptional adaptation is transient and replicative parasites recovered from PAT (POST-PAT) exposure have an almost identical transcriptional profile as their No PAT counterparts, as evidenced by an extremely low number of DE genes (4 and 33, (|log2FC|≥1, adjpvalue > 0.05 for BPK275 and BPK026, respectively, Supplementary Table 4).

### Transition through PAT induced quiescence induces hyper-resistance in *L. donovani* lines with pre-existing PAT resistance

Although the quiescent state was found in all but one of the studied lines, the different killing profiles observed earlier suggested different survival mechanisms. Specifically, P1 parasites seemed to behave similarly to classical bacterial persisters with no growth during exposure to the drug, while P3 and P4 parasites were able to resume growth even under drug exposure after a short transition through the quiescent state, suggesting that a small subpopulation of these resistant parasites became hyper-resistant. To ascertain whether drug-pressure was selecting for altered genome sequences of parasites isolated post-exposure to PAT, we analysed the PAT-recovered parasites with particular focus on their PAT susceptibility and potential genomic alterations.

First, we measured the PAT susceptibility of the cells that recovered from the PAT exposure by comparing the IC_50_ without drug exposure (No PAT) and after removal of PAT and recovery of growth (Post-PAT) (Table 1). As reported previously, we found that the P1 parasites (ISC1 group) showed the highest susceptibility, with IC_50_ values ranging between 9 ± 0.6 and 19 ± 1.2 µM. The P3 and P4 parasites exhibited a lower susceptibility, with IC_50_ values ranging between 46.4 ± 0.5 µM and 137.1 ± 11.9 µM. When control and Post-PAT conditions were compared, two patterns were observed: (i) P1 lines did not show changes in IC_50_ after the exposure to PAT; (ii) in contrast, the P3 and P4 lines showed a significant increase of IC_50_ in Post-PAT conditions. The result observed for the P1 parasites is consistent with the classical behaviour reported for persister cells in bacteria. However, the extreme increase in IC_50_ values in such a short period of time observed for P3/P4 parasites was unexpected, but consistent with the ability of these parasites to grow in the presence of PAT in concentrations exceeding those which had prevented growth prior to the adoption of a quiescent state.

**Table 1:** List of *L. donovani* strains and their susceptibility to trivalent antimony (PAT)

Strain	ISC population	H-locus amplified	AQP1 indel	IC_50_ PAT No PAT	IC_50_ PAT Post PAT
**Profile 1**^**a**^					
BPK026	ISC1	No	No	9.0 ± 0.6	6.7 ± 0.7
BPK031	ISC1	No	No	19.0 ± 1.2	20.9 ± 4.6
BPK156	ISC1	No	No	18.2 ± 0.7	19.7 ± 0.1
**Profile 2**^**a**^					
BPK190	ISC4	Yes	No	nd	nd
BPK087	ISC4	Yes	No	nd	nd
LdBOB	Other	No	No	nd	nd
**Profile 3**^**a**^					
BPK085	ISC6	Yes	yes^c^	89.9 ± 0.5	241.5 ± 14.7
BPK282	ISC6	Yes	No	137.1 ± 11.9	423.5 ± 2.8
BPK294	ISC9	Yes	No	40.2 ± 4.4	230.1 ± 13.9
**Profile 4**^**a**^					
BPK080	ISC4	Yes	No	52 ± 1.7	86.8 ± 2.6
BPK275	ISC5	Yes	Yes^b^	46.4 ± 0.5	214.1 ± 30.1

Cloned strains of *Leishmania donovani* isolates from the Indian Subcontinent and reference strain (LdBOB) with their respective drug susceptibility in promastigotes (IC_50_, µM). The values were measured by resazurin assay after removing normally lethal PAT dose, 10-day (post-PAT); and the control (No PAT) was maintained in parallel and not exposed to PAT. For three lines that did not recover after drug exposure, the data is not reported and denoted as ‘n.d.’ (not determined). The data is presented as the mean ± SEM of three biological and technical replicates. The ten cloned strains of *L. donovani* were isolated in the ISC^16^, and showed different levels of susceptibility to antimony (i) three strains where no obvious genomic changes indicative of PAT resistance are known to be present (ISC1); these strains are highly susceptible to PAT; (ii) seven strains belonging to different genetic groups (ISC4, 6, and 9) characterized by possessing one known genomic marker of resistance to PAT, the intra-chromosomal amplification of the H-locus, which contains the MRPA gene, a known driver of antimony resistance; these strains show decreased susceptibility to PAT; (iii) one strain with low susceptibility has two known genomic drivers of PAT-resistance: a 2-nt insertion (458_459dupTC|p.Ala154fs) that introduces a stop codon in the *aqp*1 gene^16^, proposed to be associated with antimonial resistance observed in the ISC5 genotype in addition to the intra-chromosomal amplification of the H-locus; the strain shows a low susceptibility to PAT^16,53^. We also included a reference *L. donovani* strain isolated in East Africa (LdBob), highly susceptible to antimony (Table 1)^54,55^. Of note, one of the strains belonging to ISC6 group, BPK085, also carries a different 2-nt insertion mutation (94_95dupAA|p.Asn32fs) in the *AQP1* gene, which results in a frameshift^16^.

^a^Profiles 1–4 correspond to patterns of survival under normal lethal PAT dose.

^b^2-nt Indel in position 458_459dupTC|p.Ala154fs of the *AQP1* locus, causing a stop-codon, present in all ISC5 isolates^16^.

^c^2-nt indel in position 94_95dupAA|p.Asn32fs of the *AQP1* locus.

As the main genomic difference between the P1 and P3/P4 lines is the increased copy of the *MRPA*, a membrane ABC transporter known to be involved in resistance to antimony, we investigated whether increased levels of MRPA could underlie the observed reduction in PAT susceptibility in Post-PAT P3/P4 parasites. To address this, we made use of transcriptomic data generated for one P1 and P4 line (BPK026 and BPK275, respectively) described above. As expected, we found *MRPA* transcript to be more abundant in BPK275, but no further changes were observed during and post-PAT exposure (Supplementary Fig. 5). Next, we decided to assess the expression of other members of the ABC protein family, which were initially described in *L. major* and of which some were shown to be linked to drug resistance^34,35^. Although we observed difference in transcript abundance for several ABC transporters between BPK275 and BPK026 at different time points, no clear increase in transcript levels of the ABC transporters in Post PAT BPK275 could be detected (Supplementary Fig. 5). Together with negligible transcriptional differences between BPK275 no PAT and post PAT (4 DE genes, (|log2FC|≥ 1, adjpvalue > 0.05), this suggests that phenomena other than transcriptional adaptations are responsible for the increase in IC_50_ after the transition through drug-triggered quiescence in the P3/P4 parasites.

**Fig. 4:**
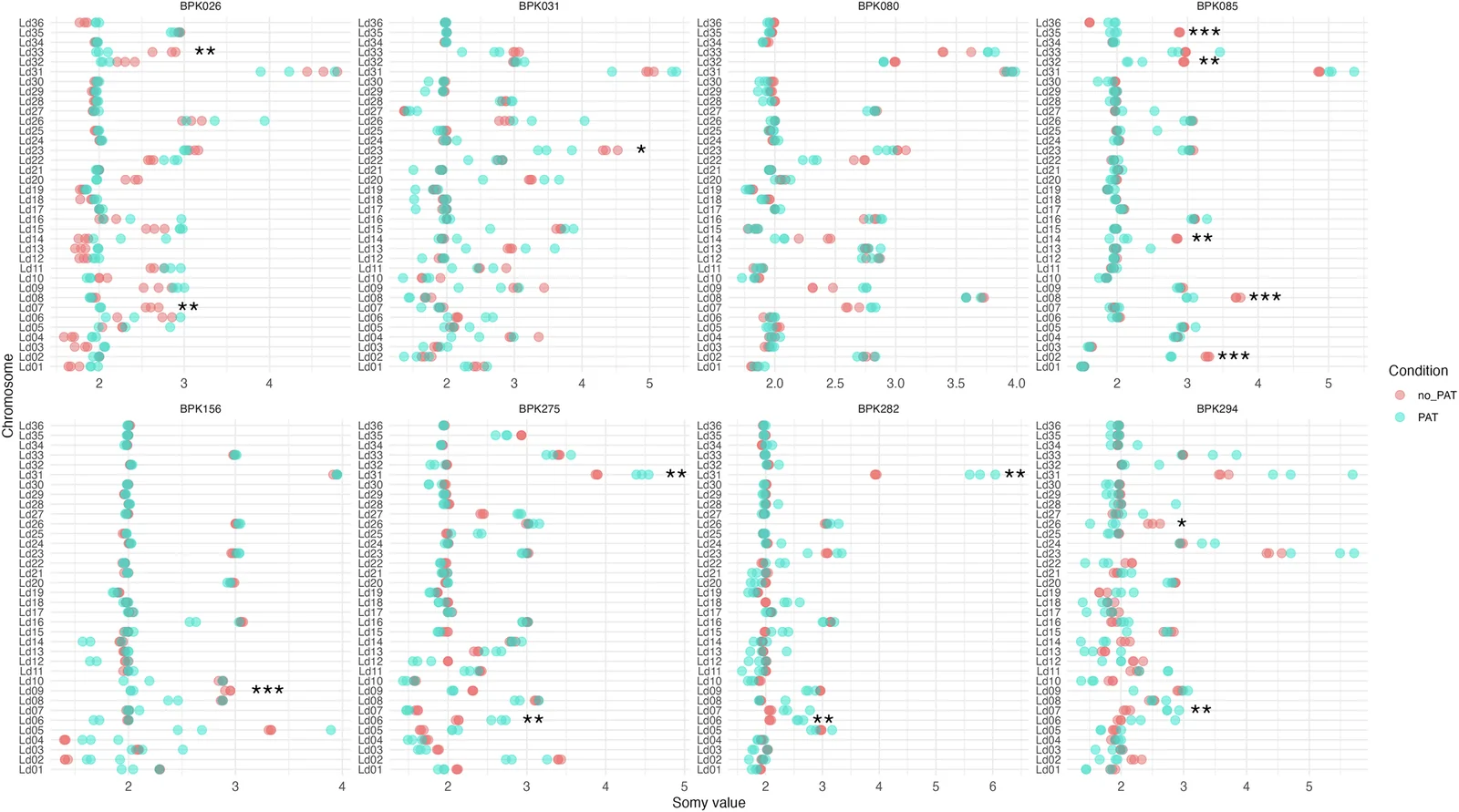
Dot plot containing the somy values per chromosome and per strain. For each chromosome and each strain, the somy value is indicated in the non-exposed samples (red, No_PAT) and the PAT exposed sample (green, PAT). Statistically significant somy differences using student *t*-test between non-exposed versus PAT-exposed samples are indicated by asterisks per chromosome and strain (* < 0.05, ** < 0.01, *** < 0.001). The data represent the values of three biological replicates.

We then sequenced (genome sequencing statistics in Supplementary Data 6) and compared the genome of control and Post-PAT, in triplicates, in order to search for possible new signatures of drug resistance. In our analysis, we focused on potential changes of SNPs, INDELs, copy number of individual genes and the H-locus and somy values. We did not observe consistent genomic alterations in either P1 or P3/P4 Post-PAT parasites when SNPs, INDELs, and depth of individual genes, including *MRPA* were analysed (see details in supplementary text, Supplementary Figs. 6–8, Supplementary Table 5, and Supplementary Data 7). We observed a general decrease of somy of several chromosomes in both P1 and P3/4 lines in Post-PAT parasites as compared to non-exposed lines (Fig. 4 and Supplementary Fig. 9). However, no consistent changes in copy number variation of individual chromosomes in either P1 or P3/P4 Post-PAT parasites could be detected.

In summary, no consistent genomic alterations could be detected in either P1 or P3/4 parasites. This further supports persister-like nature of a small population in P1 parasites that survive normally lethal doses of PAT. In the case of P3/P4 parasites, we report here detection of a unique form of transient persisters with the ability to retain their reduced PAT susceptibility in drug-free conditions, showing no discernible genetic change when compared to the precursors. Further investigation is required to ascertain the mechanism allowing the growth in PAT and increase of IC_50_ in recovered P3/P4 parasites.

### PAT-tolerant promastigotes show cross-tolerance to other drugs

**Fig. 5:**
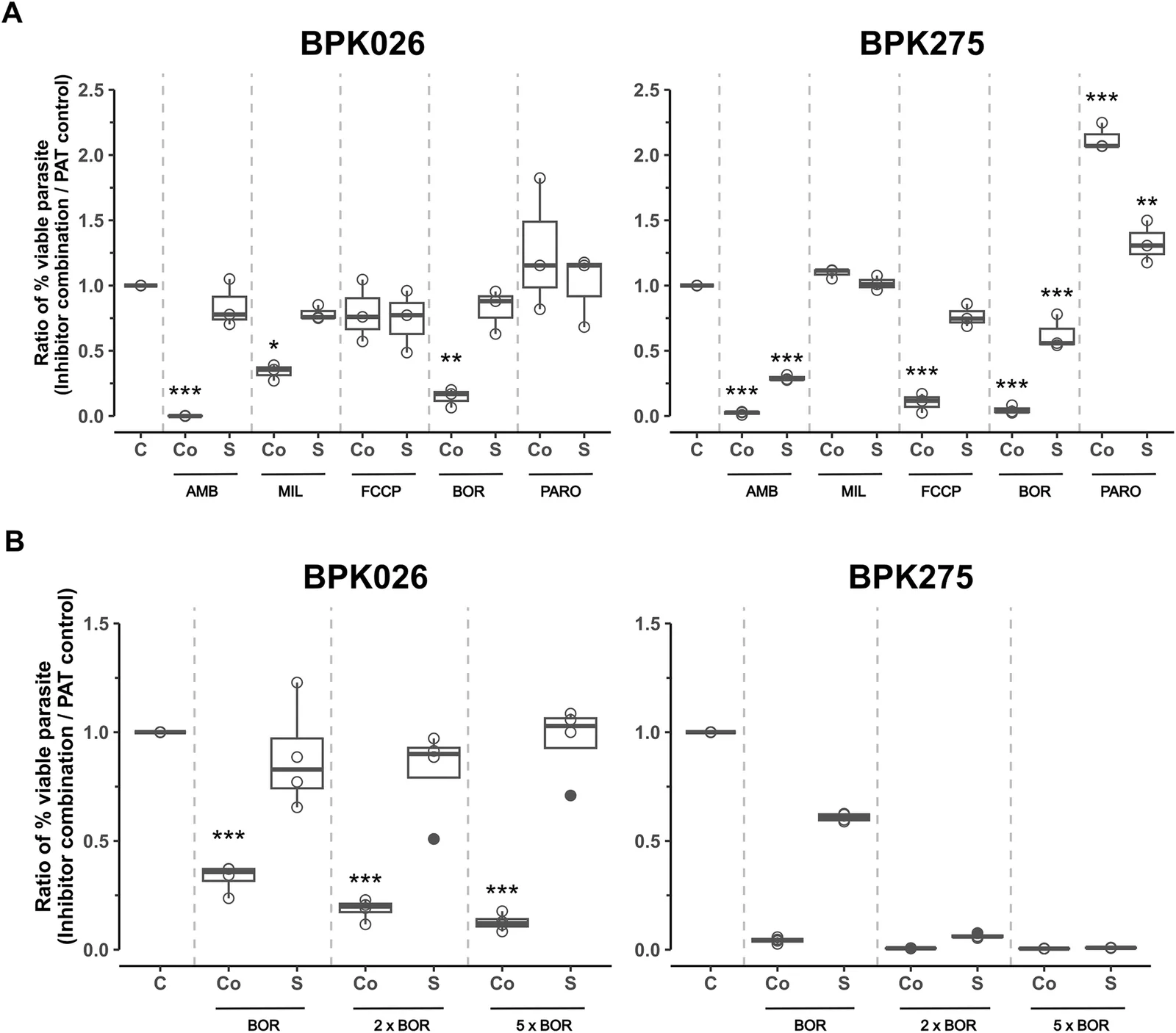
Survival of PAT-tolerant *Leishmania* promastigotes when exposed to other drugs. Drugs with different mode of action were used: amphotericin B (AMB), miltefosine (MIL), bortezomib (BOR), carbonycil (FCCP) and paromomycin (PARO). Briefly, (i) AMB binds ergosterols and potentially destabilises membranes, (ii) MIL is a lipid binding compound and likely also acts through destabilization of membranes, (iii) BOR is a proteasome inhibitor, (iv) FCCP is a protonophore, which can permeabilise the mitochondrial membrane, and (v) PARO is a probable protein synthesis inhibitor^36–40^. The drugs were applied at their respective IC_50_ concentrations in both Co and S conditions. As a control exposure, we repeatedly treated with PAT during the first and second exposure (Supplementary Table 7). To assess the killing effect of these drugs, we quantified the % of viable promastigotes in relation to the control exposure (C). **A** Ratio of percentages of viable parasites submitted to different compounds (IC_50_) vs normally lethal doses of PAT (control, C) and following two drugging schemes (Co, combination and S, serial); (i) MIL, Miltefosine; AmB, Amphotericin B, PARO, Paromomycin; BOR, Bortezomib and FCCP, Carbonycil. The data is shown as box plots of three biological replicates for each combination or serial, with the corresponding individual values represented as circles. **B** Ratio of percentages of viable parasites submitted to BOR (1x IC_50_, 2x and 5x IC_50_) vs normally lethal doses of PAT (control, C) and following two drugging schemes (Co and S). The data is shown as box plots of four biological replicates for each combination or serial, with the corresponding individual points represented as circles. The asterisks indicate statistically significant differences for each scheme compared to the control, analyzed by Tukey’s post-hoc test (**A**) and two-tailed Welch’s two-sample *t*-test, (**B**)*P < 0.05, **P < 0.01, ***P < 0.001.

In bacteria, entering into a non-proliferative physiological state after the exposure to one drug can provide tolerance to other antibiotics^6–8^. Hence, we tested whether the observed PAT tolerance, associated with the acquired quiescent state in the BPK026 and BPK275 lines, would also provide tolerance to other known antileishmanial compounds. We, therefore, evaluated the susceptibility of PAT-tolerant promastigotes of both BPK026 and BPK275 towards 5 compounds with different modes of action (see legend Fig. 5)^36–40^. First, we found that the IC_50_ values for each of these compounds were comparable in BPK026 and BPK275 (Supplementary Table 6), except for PARO, to which BPK275 was three-fold less susceptible. To assess the protective effect of PAT-associated tolerance, we first triggered quiescence by exposing the promastigotes to PAT (strain-specific normally lethal doses) for 5 days, followed by 5 days of simultaneous exposure of PAT and the additional drug (serial exposure (S), Supplementary Table 7). If the viability ratio for a given drug was similar to that of PAT, it would mean -in serial exposure- that PAT-induced quiescence provides cross-tolerance to the second drug. Secondly, we added both PAT and the second drug from the very beginning (combination exposure (Co), Supplementary Table 7). In that case, a decrease of the viability index of the combination would be due to the additive effect of the drugs (in that experimental scenario, there is no time to induce quiescence with PAT).

In the case of BPK026, two main patterns were observed. On one hand, with AMB, MIL, and BOR, there was a strong reduction of the viability ratio in respective combination exposures (0.001 to 0.34 range), while in the serial exposures, the viability was comparable to the levels in control treatment (0.79-0.82 values in S, and 1.0 in C; Fig. 5A). This indicates that PAT-triggered BPK026 quiescent cells gain tolerance to AMB, MIL, and BOR. On the other hand, with FCCP and PARO, viability remained similar to control exposure in both Co and S conditions, suggesting that the used concentrations of FCPP and PARO were too low to enhance PAT killing (Fig. 5A). Results were confirmed by the reversibility assay: *i.e*. the combination of PAT + AMB for which growth was observed in only one out of three replicates, while all three replicates demonstrated proliferation in the case of serial exposure (Table 2). In the case of BPK275, results were different and 5 patterns were observed (Fig. 5A): (i) for BOR, a low viability ratio in Co (0.047 ± 0.02 value), and comparable viability in the C exposure and S exposures, suggesting cross-tolerance to BOR in PAT-exposed quiescent cells, (ii) lower viability ratios for AMB (both Co and S exposures) in comparison to C, but the viability in S was significantly higher than in Co, suggesting some level of protection against AMB provided by quiescence of PAT-exposed cells (iii) same viability as control for MIL (both Co and S conditions), indicating that the used MIL concentrations were too low to enhance PAT killing, (iv) for FCCP, although low viability in S was observed, microscopic examination revealed an abundance of parasites, suggesting that parasites likely died due to nutrient deprivation, and (v) for PARO a higher viability ratio in Co exposure in comparison to C and a ratio close to the control under serial exposure, suggesting that PARO and PAT act synergistically to trigger the presence of quiescent cells, but also a result that could be partly explained by the low susceptibility of BPK275 to PARO. In all conditions, the reversibility assay revealed proliferation in the 3 replicates (Table 2). Collectively, these results show that PAT-tolerant cells also show tolerance to other drugs, but the degree of this cross-tolerance is drug- and strain-dependent.

**Table 2:** Reactivation of parasite proliferation (numbers out of 3 or 4 replicates) after exposure to a combination of PAT and another drug or serial drugging (see experimental design in Supplementary Tables 7 or 8)

Condition	Drug/Inhibitor	P1	P4	Drug/Inhibitor	P1	P4
		BPK026	BPK275		BPK026	BPK275
Control	PAT	3 of 3	3 of 3	PAT	4 of 4	4 of 4
Combination	AMB	1 of 3	3 of 3	BOR	0 of 4	4 of 4
Serial		3 of 3	3 of 3		0 of 4	4 of 4
Combination	MIL	3 of 3	3 of 3	2 x BOR	0 of 4	2 of 4
Serial		3 of 3	3 of 3		1 of 4	4 of 4
Combination	FCCP	2 of 3	3 of 3	5 x BOR	0 of 4	0 of 4
Serial		1 of 3	3 of 3		1 of 4	4 of 4
Combination	BOR	0 of 3	3 of 3			
Serial		1 of 3	3 of 3			
Combination	PARO	3 of 3	3 of 3			
Serial		3 of 3	3 of 3			

To analyze the cross-tolerance further, we focused on protection against BOR, for which we also executed combination and serial exposure and compared the effects of applying BOR at 3 different concentrations: at 1x, 2x, and 5x IC_50_ of BOR (see experimental design in Supplementary Table 8). In the case of BPK026, viability ratios slightly decreased with the increasing concentration of BOR in the combination, condition, but this did not affect the observed cross-tolerance in S exposure (Fig. 5B). In contrast, in BPK275, viability ratios decreased with the increase of BOR concentration in both conditions, with almost no viable cells detected in the two highest concentrations of BOR (Fig. 5B). The reversibility assays showed that none of the Co replicates could grow, while a few replicates in the S condition could re-proliferate in BPK026. By contrast, for BPK275, all replicates in the S condition could reactivate, while only two or none were able to proliferate in the higher concentrations of BOR in Co exposure (Table 2). This indicates that cross-tolerance to BOR could be observed for both strains, but it was more pronounced for the antimony resistant BPK275 line.

## Discussion

This study demonstrates that genetically different *L. donovani* strains use quiescence as a survival strategy under normally lethal doses of trivalent antimony. In most lines, upon exposure to PAT a small non-replicating population with reduced metabolism was observed and had the ability to survive the treatment. Quiescence was detected in all parasites with pre-existing genomic signatures of PAT resistance, and these cells could also resume growth while still under treatment exposure, enhancing the level of resistance to PAT in so doing. Quiescence, therefore, appears to be a widespread phenomenon in *Leishmania* parasites exposed to normally lethal doses of a drug.

Here we triggered entry into quiescence by exposure to antimony, analogous to triggered persistence in bacteria rather than spontaneous persistence^4^. All lines surviving normally lethal doses of PAT showed a bi-phasic time-kill curve typical for bacterial persister cells^41,42^, though in two lines, BPK275 and BPK080, the subpopulation of PAT tolerant cells was larger than typical. Detailed analysis of the lines with the most extreme survival phenotypes revealed several properties typical of persisters, including reduction of metabolism, proliferation and mitochondrial membrane potential with no apparent genomic changes and cross-tolerance to other antileishmanial drugs. However, the ability to resume growth after a transient adoption of quiescence observed in lines with pre-existing antimony resistance is inconsistent with classical definition of bacterial persisters, since these lines acquire a further reduction in sensitivity to drug during the passage through quiescence. Because of this unique mixture of observed phenotypes in the quiescent *L. donovani* lines, we collectively call them persister-like cells.

Regardless of the presence of genomic drug resistance, we observed strong downregulated transcription of core metabolic pathways, with the effect being more pronounced in the PAT sensitive BPK026. At the same time, the detected upregulation of autophagy is one of the conserved features found across other quiescent eukaryotic cells^10^, including a dermotropic *Leishmania* species, *L. lainsoni*^23^. The identification of numerous upregulated genes, and in particular those encoding proteins of unknown function (Supplementary Table 4), opens a possibility for discovery of *Leishmania*-specific biomarkers or regulators of a persister-like state.

Parasites without known antimony resistance, when triggered to enter quiescence over a 10-day period, remain quiescent while still under drug treatment and resume growth only when transferred to antimony-free medium. By contrast, *Leishmania* that had harboured genetic mutations that yielded resistance to antimony also entered quiescence when initially exposed to antimony at a dose beyond that to which they are normally resistant. However, these cells were able to re-enter growth phase whilst in medium containing drug. Remarkably, these parasites that resume growth whilst still in medium containing antimony are actually more resistant to the drug than they were before having passed through quiescence. Although we do not have a molecular explanation for this, we demonstrated that this hyper-resistant phenotype is not underlined by consistent genomic changes, general transcriptional remodelling or an increase in the transcript levels encoding MRPA or related ABC transporters. As the gene encoding the MRPA pump, which removes antimony from the cells^43^ is already amplified as part of the H-locus in resistant cells, we cannot exclude its levels are further increased through post-translational modifications in these strains. Translational reprogramming previously reported as a mechanism of antimony resistance^44^ could also drive the observed hyper-resistant phenotype. It will be interesting to explore this in future work.

We also tested the ability of cells in which quiescence was triggered by PAT to tolerate exposure to a range of other drugs. By adding 5 other compounds, with different modes of action, at their IC_50_ levels, alongside PAT on day zero, we were assessing their toxic effect on non-quiescent cells. By contrast, by adding the secondary drugs five days after the addition of PAT, we were testing their ability to kill pre-selected quiescent cells. AMB, MIL and BOR had significantly reduced ability to clear cultures if added after 5 days, while this was not the case for PARO and FCCP. Mechanistically, we had assumed AMB might not have a different bearing on quiescent cells as it impacts membranes after binding ergosterol. However, quiescent cells appear to be less sensitive to the drug, possibly indicating membrane remodelling during quiescence, which might also explain why quiescent cells also showed tolerance to MIL. In the case of the proteasome inhibitor BOR, the general decrease in protein turnover in quiescent cells may explain why cross-tolerance is seen to this compound. Of note, BOR, as a proteasome inhibitor, has a similar mode of action as LXE408, a compound well advanced in clinical trials for release as a new therapy for VL^45^, indicating a risk of similar cross-tolerance issues in the future if used in patients carrying quiescent persister-like cells. FCCP interferes with proton-dependent transport processes at the mitochondrial and plasma membranes, and given the requirement to sustain key metabolic processes, the fact that quiescent cells retain sensitivity was not a surprise. As an inhibitor of protein synthesis, paromomycin likely continues to act on processes crucial to the viability of quiescent cells, which would explain the absence of cross-tolerance to this drug. However, this last result could be biased by the intrinsic low susceptibility of BPK275 to paromomycin. Cross-tolerance to several antifungal compounds has been observed in pathogenic fungi and was linked to changes in aneuploidy patterns and not the persister state^46,47^. This was not observed here, but given the aneuploidy dynamics and mosaicism in *Leishmania*^18,48,49^ we cannot exclude that some chromosome copy number changes contributed to the detected cross-tolerance in persister-like cells.

This study was carried out with the extracellular promastigote form of *Leishmania* and should be repeated with both axenic and intracellular amastigote forms to confirm the same phenomena are applicable to the clinically relevant stage. Several studies have already indicated the existence of persister-like cells in *Leishmania* infections of mammals, and it seems increasingly likely that *L. donovani* persister-like cells can explain some cases of treatment failure in the absence of drug resistance and post-kala azar dermal leishmaniasis (PKDL), observed years after clinical healing of VL.

In summary, this report demonstrates a worrying, intrinsic ability of *Leishmania* to tolerate high lethal doses of a drug and exhibit cross-tolerance to different drugs, even with non-overlapping modes of action. We also unravelled unexpected effects of adoption of quiescence, which may go beyond the period of exposure to drug. In strains with pre-existing drug resistance, transition through the quiescent state potentially facilitates the development of a form of population-wide super drug resistance. Thus, even a short period of quiescence may have much more serious impact than previously thought, with a potential to create super-resistant parasites. Further work is required to verify our observations in an intra-macrophage context, both in vitro and in vivo.

## Methods

### Parasites

Eleven strains of *L. donovani* were used in this study (Table 1). Ten were derived from clinical isolates originating from VL patients in Nepal and classed into 3 main genotypes according to the amplification or not of H-locus (containing *MRPA*) and *AQP1* sequence^16^ : (i) No amplification of H-locus and wild type *AQP1* (H−/AQP+): MHOM/NP/2002/BPK026/0 clone 5 (LdBPK026), MHOM/NP/2002/BPK031/0 clone 2 (LdBPK031) and MHOM/NP/2002/BPK156/0 (LdBPK156). (ii) Amplification of H-locus and wild type *AQP1* (H+/AQP+): MHOM/NP/2002/BPK080/0 clone 1 (LdBPK080), MHOM/NP/2002/BPK085/0 clone 8 (LdBPK085), MHOM/NP/2002/BPK087/0 clone 11 (LdBPK087), MHOM/NP/2002/BPK190/0 clone 3 (LdBPK190), MHOM/NP/2003/BPK282/0 clone 4 (LdBPK282), MHOM/NP/2003/BPK294 clone 1 (LdBPK294). (iii) Amplification of H-locus and *AQP1* indel in position Ld31_0007735 (H+/AQP−): MHOM/NP/2003/BPK275/0 clone 18 (LdBPK275). A last strain originating from Sudan was used as a reference: MHOM/Sudan/1962/1S-2D Bob (LdBOB). All strains were cryopreserved at the Institute of Tropical Medicine in Antwerp, Belgium. Promastigotes were maintained at 26 °C in M199 medium (Thermo Fisher) at pH 7.2 supplemented with 20% of heat-inactivated fetal bovine serum (HI-FBS), 5 mg/mL hemin, 10 µM acid folic, 1X RPMI vitamin mix, 2 mM glutamine, 100 units/mL of penicillin, and 100 µg/mL of streptomycin, which was referred to as M199-sup in the following experiments. For transfections, HOMEM medium (Gibco) supplemented with 20% HI-FBS, referred to as HOMEM-sup, was used to transfect *Leishmania* promastigotes mentioned below.

### Drugs

The drugs used in this study were: (i) for leishmaniosis treatment: potassium antimonyl tartrate (383376, Sigma-Aldrich), miltefosine (M5571, Sigma-Aldrich), amphotericin B (A2942, Sigma-Aldrich), and paromomycin (P5057, Sigma-Aldrich); (ii) in development: bortezomib (5.04314, Sigma-Aldrich), and carbonyl cyanide 4-(trifluoromethoxy) phenylhydrazone (C2920, Sigma-Aldrich). The stock solutions of potassium antimonyl tartrate (PAT), paromomycin (PARO), and miltefosine (MIL) were dissolved in PBS, meanwhile, bortezomib (BOR) and carbonyl cyanide 4-(trifluoromethoxy) phenylhydrazone (FCCP) in dimethylsulfoxide (DMSO) at a concentration of 20–50 mM and stored at −20 °C. The working solutions were freshly diluted in PBS or dimethylsulfoxide (DMSO) and added to M199-sup until reaching the desired final concentrations for the different drugs/inhibitors. A control containing either PBS (0.5% v/v) or DMSO (0.1% v/v), depending on which was used to dissolve the drug or inhibitor, was included in the plate. The addition of PBS or DMSO did not interfere with the parasite growth.

### Engineering of fluorescent lines

For each strain, the enhanced green fluorescent protein (EGFP) was integrated within the 18S ribosomal DNA locus (rEGFP) of promastigotes, with pLEXSY-hyg system (Jena Bioscience). rEGFP was previously validated as a negative marker for quiescence: highly expressed in proliferative cells and downregulated during quiescence^29^. For transfection, *L. donovani* promastigotes were maintained at 26 °C in HOMEM-sup with passages each 4–7 days at a dilution 1/50. A quantity of 2 × 10⁷ promastigotes was transfected with 100 ng of the linearized DNA fragment containing EGFP, using the Basic Parasite Nucleofector kit 1 (VMI 1011, Lonza); program U-033. After 24 h of transfection, promastigotes harboring rEGFP were selected with hygromycin B at a final 25 µg/mL concentration. Parasites were passaged 3 times in HOMEM-sup with hygromycin B until the isolation of individual clones. An appropriate dilution of 2-day promastigotes culture was washed with PBS and adjusted to a concentration of 2 × 10^6^ parasites/mL in PBS containing 2% HI-FBS, 100 units/mL of penicillin, and 100 µg/mL of streptomycin. Then, the suspension was filtered using a 5 µM pore filter to remove cell clumps. A total of 8 individual clones were sorted by fluorescence-activated cell sorting (FACS), using S3e TM Cell Sorter (BIORAD) using a 488 nm laser for EGFP excitation. Single cells expressing EGFP were selected based on FSC-A vs SSC-A followed by FSC-A vs FSC-H, and FSC-A vs EGFP, and collected into 300 µL of HOMEM-sup. The clonal expansion was monitored for two weeks until enough promastigotes were present for further passages. The EGFP fluorescence of the clones was assessed by FACSVerse cytometer (BD Biosciences), where the clones with more than 99% of cells expressing EGFP and the highest median fluorescence intensity (MFI) were selected for further experiments. The generated lines were called rEGFP lines, maintained in M199-sup, and then cryo-preserved.

### Estimation of PAT concentration at which parasites reach a minimal metabolic activity

Promastigotes of the *L. donovani* rEGFP lines were synchronized by consecutive passages every two days to ensure that the promastigotes were in the proliferative phase. Then, parasites were distributed (5 × 10⁵ parasites/mL) in 200 µL of M199-sup in sterile 96-well culture plates and exposed to different concentrations of PAT ranging from 1200 to 12.5 µM for 4 days at 26 °C. An additional well was included with only M199-sup as a control for each *L. donovani* line. Resazurin sodium salt (Sigma) was added at a final concentration of 80 µM and incubated for 5 h in the dark at 26 °C. The fluorescence was measured using the Victor X2 Multilaber Reader (PerkinElmer) with a 560-nm excitation and 590-nm emission wavelength. The fluorescence measurements for each line were normalized in comparison to their respective control, with only M199-sup to calculate the percentage of cells with metabolic activity. The lethal PAT concentrations for each line were determined as the drug concentration point where the percentage of metabolic active cells was less ~1%.

### Monitoring quiescence and cell viability during PAT exposure

The rEGFP lines were passaged twice each 2 days to synchronize them at the logarithmic phase, and then a density of 5 × 10⁵ parasites/mL was exposed to a normally lethal PAT doses during 10 days at 26 °C, where M199-sup with PAT was refreshed once after 5 days. The respective controls were prepared for each line with only M199-sup, and promastigotes were collected daily. At each time point, parasites were centrifugated at 1800 × *g* for 10 min, resuspended in PBS with 1 µM NucRed Dead 647 (Thermo Fisher), a dead marker that binds to DNA when the cell membrane is damaged and emits fluorescence. After 10 minutes at room temperature, the samples were analyzed by FACSVerse (BD Biosciences) cytometer including as controls: (i) negative control, wild-type line, and not-stained, and (ii) a dead control, parasites exposed to thermal shock at 70 °C for 10 min. The gating scheme (Supplementary Fig. 10) was initiated with a large gate of FSC-A vs SSC-A to include all cells due to the heterogeneous size and complexity of the parasites, continued with FSC-H vs FSC-A gate to avoid doublets or aggregates. A gate of FSC-A vs NucRed Dead (−) was used to exclude dead cells, and live cells were determined based on a gate of NucRed Dead (−) vs EGFP(+). The Flow Cytometry Standard (FCS) data files were analyzed with the FlowJo™ v10.10 to determine the median fluorescence intensity (MFI) of EGFP and the percentage of viable cells.

### Reversibility test

Promastigotes from the different *L. donovani* rEGFP lines under PAT exposure during all the combination and serial conditions were harvested and washed once with M199 medium by centrifugation at 1800 × *g* for 10 min. The supernatant was removed without disturbing the pellet, resuspended in M199-sup, and incubated at 26 °C. Then, we monitored the cultures weekly for 21 days under microscopic observation until flagellated mobile promastigotes were observed.

### Promastigote susceptibility assay

Susceptibility of the promastigotes was assessed in the eight lines that survive PAT exposure and revert to a proliferative state, including their respective controls maintained in only M199-sup medium. Likewise, the promastigotes of BPK026 and BPK275 were used to measure the susceptibility against five drugs for the cross-tolerance experiments. The measurement was performed using the resazurin method as previously described with minor modifications^19,50^. Briefly, logarithmic-stage promastigotes (5 × 10⁵ parasites/mL) were plated in 200 µL of M199-sup in sterile 96-well culture plates and grown in the presence of M199-sup alone (control) and medium containing serial dilution of drugs for a period of 72 h at 26 °C. Then resazurin sodium salt (Sigma) was added at a final concentration of 80 µM, and the parasites were incubated for 5 h at 26 °C, protected from light. The fluorescence was recorded with an excitation/emission 560/590 nm using the Victor X2 Multilabel Reader (PerkinElmer). All experiments were done with three independent cultures with two or three technical replicates each. The IC_50_ (50% inhibitory concentration measurement) was calculated with GraphPad Prism 8 using a sigmoidal dose-response model with variable slope.

We measured the PAT susceptibility of the cells that recovered from the PAT exposure by comparing the IC_50_ without drug exposure (No PAT) and after removal of PAT and recovery of growth (Post-PAT). The post-PAT strains were transferred to M199-supplemented medium without drug pressure and maintained until recovery was microscopically confirmed by the presence of motile promastigotes. The recovery duration varied from 0 days (BPK275, which was already proliferating) to 5 weeks (BPK026). Once microscopically visible parasite growth was observed, the parasites had one passage in M199-supplemented medium for 3 days (showing normal parasite growth) before IC_50_ measuring.

### DNA preparation and bulk whole-genome sequencing (WGS) analysis

Promastigotes were harvested from the eight lines that reverted to a proliferative state after PAT exposure or controls maintained without it. The parasites were washed with PBS and the genomic DNA was extracted using the QIAamp DNA Mini Kit (51306, Qiagen) following the manufacturer’s recommendations. The DNA concentration was measured using the Qubit dsDNA Quantification Assay Kit broad-range (Q32853, Thermo Fisher). The libraries PCR-free were prepared and sequenced on the Illumina NovaSeq6000 platform using 2 × 150 bp paired-end reads aiming for a sequence depth of ~100x for each sample, a service provided by GenomeScan (Netherlands).

After quality control of the raw fastq files using FastQC, the reads were mapped to the *L. donovani* reference genome LdBPK282-V2^18^, using BWA v0.7.17 using a seed length of 50. Reads with a mapping quality higher than 30 were selected using SAMtools, duplicate reads removed using the Picard MarkDuplicates command (version 2.22.4), and only properly paired reads were retained using samtools. The somy values were estimated as the median reads count per million (CPM) per 10 kb bin of chromosomes normalized by the median CPM per bin of 5 chromosomes surrounding on both sides. SNP calling was conducted using the Genome Analysis Toolkit (GATK) pipeline (version 4.1.4.1) following GATK’s best practices guidelines. The process included: (1) generating GVCF-formatted files using GATK HaplotypeCaller, (2) combining the GVCF files from all samples with the GATK CombineGVCF command, (3) performing genotyping with the GATK GenotypeGVCF command, and (4) filtering SNPs and indels based on GATK’s recommended best practices using the SelectVariants and VariantFiltration commands. For the CNV detection, for every sample, the sequencing depth per position was derived using the SAMtools depth command. The CNV value was calculated by taking the ratio of the median coverage per gene over the median coverage of the chromosome the gene is located on. Statistical tests comparing either CNV or Somy values were performed using a student’s *t*-test. Visualization of somy values, SNP data and CNV values was performed in R (version 4.2.0) using heatmaps and dot plots. Heatmaps were created with the pheatmap package (version 1.0.12), while dot plots were generated using the ggplot2 package (version 3.4.1).

### Purification of alive promastigotes by Percoll density gradient

The live *Leishmania* parasites were separated from dying cells under PAT exposure using a Percoll gradient as previously reported. In short, promastigotes were harvested, resuspended in 5 mL of M199, and passed through a 26 G needle 4 times to disrupt cellular aggregates. The suspension was centrifuged at 1800 × *g* for 10 min, and the pellet was resuspended in 6 mL of 45% Percoll solution in PBS. Then, the discontinuous gradient was prepared based on 1.5 mL of 70% Percoll at the bottom, followed by a slow addition of the suspension of parasites diluted in 45% Percoll, and 2 mL of 25% Percoll at the top. The tubes with the gradient were carefully placed without mixing the layers and centrifuged at 2300 × *g* for 45 min at 4 °C. Live promastigotes from the fraction of 45% Percoll and the interface with the 70% Percoll fraction were recovered with a pipette and washed with 10 mL of cold PBS. After resuspension in 1 mL of cold PBS, parasites were counted, and aliquots were prepared for RNA extraction. The samples were stored at -80 °C for subsequent steps.

### RNA extraction and bulk transcriptomic analysis

Promastigotes of *L. donovani* were harvested for BPK026 and BPK275 strains under different conditions: (i) parasites at the logarithmic growth phase without drug pressure (No PAT D2) for both strains, (ii) parasites exposed to normally lethal doses of PAT for 2 days (PAT D2), 50 µM or 200 µM, BPK026 and BPK275, respectively), and (iii) parasites at the logarithmic growth phase after the recovery period in M199-sup following PAT exposure at normally lethal doses for both strains (Post PAT). The samples were prepared in triplicate or quadruplicate. A quantity of 1.5 × 10⁷ promastigotes was collected, purified with Percoll gradient, and washed with PBS, as mentioned above. The total RNA was extracted using the Quick-RNA Microprep Kit (R1050, ZYMO RESEARCH) following the manufacturer’s instructions. The RNA’s quantity and quality were evaluated using the Qubit RNA Quantification Assay Kit broad-range (Thermo Fisher) and RNA Screen tape system (Agilent). The RNA-Gene Expression (poly-A selection) library preparation and sequencing service were performed by GenomeScan (Netherlands) on an Illumina NovaSeq6000 platform (2 × 150 bp paired-end reads) with a sequence depth of ~20 M PE reads/sample.

### RNA-seq bioinformatics analysis

Paired end sequencing data were aligned to the reference genome using BWA, as no alternative splicing is known in *Leishmania* species. A gene-level read count matrix was generated using htseq-count, which assigned aligned reads to genomic features based on the provided GFF annotation file. Reads were counted in a strand-specific manner using the default union mode, and only uniquely mapped reads overlapping annotated exons were retained. The resulting table of per-gene read counts served as the input for downstream differential expression analyses using DESeq2. Pairwise comparisons between conditions (No PAT, PAT D2, Post PAT) as well as between strain comparisons (BPK026 and BPK275) were obtained using the DESeq2 results function, resulting in a log₂ fold change and FDR-corrected p-values per contrast. Differentially expressed genes are defined as those genes having an absolute log₂ fold change higher than 1 and an FDR value lower than 0.05. Read-per-kilobase-per-million (RPKM) expression values were derived from the DESeq2 dataset using the fpkm function implemented in the DESeq2 package. Data visualization was carried out in R using the ggplot2 package for generating customized plots. Gene enrichment analysis was performed on multiple sets of differentially expressed genes by mapping gene identifiers to KEGG pathway annotations. Pathway over-representation was assessed using a hypergeometric test implemented in R (version 4.4.2). Resulting p-values were adjusted for multiple testing using the Benjamini–Hochberg false discovery rate (FDR) procedure. Pathways with an adjusted p-value (FDR) below 0.05 were considered significantly enriched. Enrichment results were visualized using dot plots generated with the ggplot2 package.

### Determination of mitochondrial membrane potential (MMP)

The BPK026 and BPK275 promastigotes were synchronized by two-day passaging in M199-sup before each experiment. Several cultures were prepared with a density of 5 × 10^5^ parasites/mL with normally lethal doses of PAT (50 µM for BPK026 and 200 µM for BPK275) at different times to have samples under drug exposure for 2, 4, and 10 days with their respective controls with only M199-sup. The mitochondrial membrane potential was determined using the MitoProbe™ DilC₁ (5) Assay Kit (M341151, Thermo Fisher) based on the manufacturer’s instructions. Parasites under PAT exposure were centrifuged at 1800 × *g* for 10 min, the pellet was resuspended in M199 medium and disaggregated into a single-cell suspension passing 6 times through a 26-G needle and syringe. Promastigotes were centrifuged at 1800 × *g* for 10 min, washed once in PBS, and resuspended at a concentration of 2 × 10⁶ parasites/mL in PBS containing 100 nM MitProbe DilC₁(5) for 45 min at 26 °C. Then, 1 µM SYTOX Blue Dead Cell stain (S34857, Thermo Fisher) was added to label dead cells for 5 min. In the experiment, three controls were used: (i) a dead control, heat-killed parasites at 70 °C for 10 min, (ii) a positive control, parasites treated for 10 minutes with 200 µM carbonyl cyanide m-clorophenylhydrazone (CCCP), which disrupts mitochondrial membrane potential, and (iii) a negative control, consisting of unstained parasites of a line without EGFP gene inserted. Also, fluorescence minus one (FMO) controls were used to assess autofluorescence and establish the gates for the selection of the cell populations. Subsequently, the samples were analyzed by FACSVerse cytometer (BD Biosciences) using lasers 405 nm for SYTOX Blue dye, 480 nm for EGFP, and 640 nm for MitoProbe dye. Flow cytometric analysis was performed using FlowJo™ version 10.10.

### Proliferation analysis

Approximately 1.5 × 10^7^ promastigotes from *L. donovani* BPK026 and BPK275 were labeled with CellTracker Deep Red (C34565, Thermo Fisher Technologies) following the manufacturer’s instructions. Briefly, parasites were collected, washed once with PBS, and resuspended in 1 mL of M199 medium containing 5 µM of CellTracker Deep Red. After 40 min of incubation at 26 °C, cells were washed with M199 medium, centrifuged at 1500 × *g* for 10 min, and resuspended in pre-warmed M199-sup. Following labeling, 5 × 10^5^ promastigotes/mL were treated with PAT at 50 µM (BPK026) and 200 µM (BPK275) in M199-sup or only M199-sup as a control and incubated at 26 °C protected from the light. The parasites were collected at three time points: 0, 4, and 10 days. At each measurement point, promastigotes under PAT exposure were disaggregated using a 26-G needle and syringe and centrifuged at 1800 × *g* for 10 min. The pellet and promastigotes from the controls were resuspended in PBS at a concentration of 2 × 10⁶ parasites/mL and labeled with 1 µM SYTOX Blue Dead Cell stain (S34857, Thermo Fisher) for 5 min in the dark, as a cell death marker. The fluorescence data was acquired by FACSVerse cytometer (BD Biosciences) using lasers 405 nm for SYTOX Blue dye, 480 nm for EGFP, and 640 nm for CellTracker Deep Red dye. Flow cytometric analysis was performed using FlowJo™ version 10.10.

### Cross-tolerance experiment

To evaluate tolerance to other anti-leishmanial drugs and drugs in development, of PAT-tolerant promastigotes, we exposed the promastigotes of BPK026 and BPK275 to 5 drugs with different mechanisms of action. We performed susceptibility tests by resazurin assay, mentioned above, to calculate the IC_50_ estimates. As the promastigotes were already under high stress using normally lethal PAT doses (50 µM for BPK026 and 200 µM for BPK275), we decided to use the IC_50_ estimates of each drug. We designed an experimental design consisting of two exposure times of 5 days each, for a total duration of 10 days where we had two conditions for each drug: (i) Combination, where the lethal concentration of PAT and the drug (IC_50_ estimate) were present in both exposure times, (ii) Serial, promastigotes were under normally lethal PAT concentration for the first exposure, followed by a mix of PAT with the other drug for the second exposure. As a control group, promastigotes were under only lethal PAT concentrations for both exposure times. The promastigotes were collected, centrifuged at 1800 × *g* for 10 min, and resuspended in M199 medium. The samples with large aggregates were passed through 26-G to have single-cell suspension. The parasites were centrifuged again and resuspended in PBS with NucRed Dead as a viability marker. The acquisition of the fluorescence data and its gating scheme for analysis was the same as mentioned above.

### Statistics and reproducibility

For statistical analysis on the metabolic activity, IC_50_ values, flow cytometry measurements and RNA quantity analysis, data were represented as mean values, with error bars indicating the standard error of the mean (SEM) from three or four biological replicates, as described in the corresponding Methods section and figure legends. The percentage of cells with metabolic activity was calculated using GraphPad Prism 8, which was also used to calculate the IC_50_ using a sigmoidal dose-response model with variable slope. Flow cytometry data, including MFI and viability percentage, were computed using FlowJo^TM^ v10.10. Statistical analysis for comparisons was performed in R studio (R version 4.5.1) using two-tailed Welch’s two-sample *t*-test, and one-way ANOVA followed by a post hoc Tukey’s multiple comparison test, with *p*-value < 0.05 considered statistically significant.

For the whole-genome sequencing data, comparisons of somy and CNV values between PAT-exposed and non-exposed parasites were conducted using Student’s *t*-tests based on three biological replicates per condition (PAT versus no PAT).

For the RNA-seq data, differential gene expression analyses were conducted using the DESeq2 package. Pairwise comparisons between experimental conditions and strains (biological triplicates) were performed using the DESeq2 *results* function, and resulting p-values were corrected for multiple testing using the Benjamini–Hochberg false discovery rate (FDR) procedure. Genes with an absolute log₂ fold change > 1 and an adjusted *p*-value (FDR) < 0.05 were considered significantly differentially expressed. KEGG pathway enrichment analyses were performed using hypergeometric tests in R, followed by Benjamini–Hochberg FDR correction.

For all datasets, statistical significance was indicated as *P < 0.05, **P < 0.01, ***P < 0.001. The statistical test and significance were included in the legends of the respective figures. No statistical method was used to predetermine sample size. The experiments were not randomized, and the investigators were not blinded to sample assignments during experiments and outcome assessments.

The data visualization was done using R package ggplot2 (version 4.0.0). Source data used for statistical analysis and figures are available through the Zenodo link provided in the Data availability section.

### Reporting summary

Further information on research design is available in the Nature Portfolio Reporting Summary linked to this article.

## Supplementary information

Supplementary Information

Description of Additional Supplementary Files

Supplementary Data 1

Supplementary Data 2

Supplementary Data 3

Supplementary Data 4

Supplementary Data 5

Supplementary Data 6

Supplementary Data 7

Reporting Summary

Transparent Peer Review file

## Data Availability

Raw sequencing data of the Whole Genome Sequencing data have been deposited in the Sequencing Read Archive (SRA) of NCBI under the BioProject accession number PRJNA1218669. Raw sequencing data of the RNA-seq experiment are deposited in the NCBI GEO database under the accession number GSE310227. Source data used to generate figures is stored in Zenodo https://doi.org/10.5281/zenodo.18663711 all other data are available upon request to the corresponding author.

## Code availability

Key analysis scripts used in this study for are available from https://github.com/pietermonsieurs/leishmania_q_wgs^51^ and https://doi.org/10.5281/zenodo.19675703^52^.
