# Supplementary material for: Multi-drug tolerance in Leishmania persister-like cells
Source: Commun Biol. 2026 Jun 12;9:1263. doi: 10.1038/s42003-026-10456-w (PMC13616925; doi:10.1038/s42003-026-10456-w)
Supplement: Supplementary file 1 [file 42003_2026_10456_MOESM10_ESM.pdf]

nature portfolio

Corresponding author(s): Malgorzata Anna Domagalska
COMMSBIO-25-2039-T

Last updated by author(s): Mar 17, 2025

Reporting Summary

Nature Portfolio wishes to improve the reproducibility of the work that we publish. This form provides structure for consistency and transparency in reporting. For further information on Nature Portfolio policies, see our Editorial Policies and the Editorial Policy Checklist.

Statistics

For all statistical analyses, confirm that the following items are present in the figure legend, table legend, main text, or Methods section.

n/a	Confirmed	
☐	☒	The exact sample size (*n*) for each experimental group/condition, given as a discrete number and unit of measurement
☒	☐	A statement on whether measurements were taken from distinct samples or whether the same sample was measured repeatedly
☐	☒	The statistical test(s) used AND whether they are one- or two-sided<br>*Only common tests should be described solely by name; describe more complex techniques in the Methods section.*
☒	☐	A description of all covariates tested
☐	☒	A description of any assumptions or corrections, such as tests of normality and adjustment for multiple comparisons
☐	☒	A full description of the statistical parameters including central tendency (e.g. means) or other basic estimates (e.g. regression coefficient) AND variation (e.g. standard deviation) or associated estimates of uncertainty (e.g. confidence intervals)
☐	☒	For null hypothesis testing, the test statistic (e.g. *F*, *t*, *r*) with confidence intervals, effect sizes, degrees of freedom and *P* value noted<br>*Give P values as exact values whenever suitable.*
☒	☐	For Bayesian analysis, information on the choice of priors and Markov chain Monte Carlo settings
☒	☐	For hierarchical and complex designs, identification of the appropriate level for tests and full reporting of outcomes
☒	☐	Estimates of effect sizes (e.g. Cohen's *d*, Pearson's *r*), indicating how they were calculated

Our web collection on statistics for biologists contains articles on many of the points above.

Software and code

Policy information about availability of computer code

Data collection	The FCS files were acquired by BD FACSuite v1.0.6
Data analysis	Specific software used for data analysis:<br>The inhibitory concentration measurement was calculated with GraphPad Prism 8<br>R-Package used for data analysis:<br>RStudio: v.2024.09.1<br>R-version 4.3.1<br>Mean, and standard error: stats package (base-R)<br>Student's t-test, one-way ANOVA, post hoc Tukey's multiple comparison of means test: stats package (base-R)<br><br>Key analyses scripts used in this study for the analysis of genome sequence data are available from<br>https://github.com/pietermonsieurs/leishmania_q_wgs

For manuscripts utilizing custom algorithms or software that are central to the research but not yet described in published literature, software must be made available to editors and reviewers. We strongly encourage code deposition in a community repository (e.g. GitHub). See the Nature Portfolio guidelines for submitting code & software for further information.

Data

Policy information about availability of data

All manuscripts must include a data availability statement. This statement should provide the following information, where applicable:

- Accession codes, unique identifiers, or web links for publicly available datasets
- A description of any restrictions on data availability
- For clinical datasets or third party data, please ensure that the statement adheres to our policy

- Raw sequencing data of the Whole Genome Sequencing data have been deposited in the Sequencing Read Archive (SRA) of NCBI under the BioProject accession number PRJNA1218669 (not yet released, reviewer link available via https://dataview.ncbi.nlm.nih.gov/object/PRJNA1218669?reviewer=vc5osvtljs3hep3o7ftusok877
- Raw sequencing data of the RNA-seq experiment are deposited in the NCBI GEO database under the accession number GSE310227 (publicly available).
- Source data used to generate figures is stored in Zenodo https://doi.org/10.5281/zenodo.18663711

Research involving human participants, their data, or biological material

Policy information about studies with human participants or human data. See also policy information about sex, gender (identity/presentation), and sexual orientation and race, ethnicity and racism.

Reporting on sex and gender	NA
Reporting on race, ethnicity, or other socially relevant groupings	NA
Population characteristics	NA
Recruitment	NA
Ethics oversight	NA

Note that full information on the approval of the study protocol must also be provided in the manuscript.

Field-specific reporting

Please select the one below that is the best fit for your research. If you are not sure, read the appropriate sections before making your selection.

☒ Life sciences ☐ Behavioural & social sciences ☐ Ecological, evolutionary & environmental sciences

For a reference copy of the document with all sections, see nature.com/documents/nr-reporting-summary-flat.pdf

Life sciences study design

All studies must disclose on these points even when the disclosure is negative.

Sample size	The study was based on the use of Leishmania donovani cloned isolates, 10 from Nepal, and one isolate from Sudan used as a reference. The calculation of sample size was not relevant to the study.
Data exclusions	All data were included in the analysis.
Replication	The organisms were divided into three or four biological replicates and maintained separately prior to each experiment to ensure the reproducibility of the study. All the data for each replicate is shown in each experiment.
Randomization	The independent biological replicates from each microorganism in each group were processed systematically maintaining the same conditions (reagents and equipment) to maintain the consistency in the time-series experiments. Randomization was not applicable as all isolates were subjected to DNA extraction and sequencing.
Blinding	Blinding was not implemented during data collection and data analysis. It was not relevant to this study.

Reporting for specific materials, systems and methods

We require information from authors about some types of materials, experimental systems and methods used in many studies. Here, indicate whether each material, system or method listed is relevant to your study. If you are not sure if a list item applies to your research, read the appropriate section before selecting a response.

Materials & experimental systems

n/a	Involved in the study
☒	☐ Antibodies
☒	☐ Eukaryotic cell lines
☒	☐ Palaeontology and archaeology
☒	☐ Animals and other organisms
☒	☐ Clinical data
☒	☐ Dual use research of concern
☒	☐ Plants

Methods

n/a	Involved in the study
☒	☐ ChIP-seq
☐	☒ Flow cytometry
☒	☐ MRI-based neuroimaging

Plants

Seed stocks	NA
Novel plant genotypes	NA
Authentication	NA

Flow Cytometry

Plots

Confirm that:

☐ The axis labels state the marker and fluorochrome used (e.g. CD4-FITC).

☐ The axis scales are clearly visible. Include numbers along axes only for bottom left plot of group (a 'group' is an analysis of identical markers).

☐ All plots are contour plots with outliers or pseudocolor plots.

☐ A numerical value for number of cells or percentage (with statistics) is provided.

Methodology

Sample preparation	The study included promastigotes from Leishmania donovani isolates, which were prepared according to the manufacturer's indications for each dye used, as detailed in the section Methods.
Instrument	FACSVerse (BD Biosciences)
Software	The FCS files were acquired by BD FACSuite v1.0.6<br>The FCS files were analyzed by program FlowJo_v10.10.0_CL
Cell population abundance	The selection of the population was based on a viability marker and a fluorescence marker to study quiescence developed in a previous study (Jara et al. 2019). Depending on the experiment and isolate, the percentage of viable promastigotes (our target population) varied between 0.1-92%.
Gating strategy	The gating strategy initiated with a large gate of FSC-A vs SSC-A to include all cells due to the heterogeneous size and complexity of the parasites, continued with FSC-H vs FSC-A gate to avoid doublets or aggregates. A gate of FSC-A vs NucRed Dead (-) was used to exclude dead cells, and live cells were determined based on a gate of NucRed Dead (-) vs EGFP(+). From this population was analyze the viability percentage, EGFP expression, median fluorescence intensity of mitochondrial membrane potential dye, and median fluorescence intensity of CellTracker dye.

☒ Tick this box to confirm that a figure exemplifying the gating strategy is provided in the Supplementary Information.
