# Supplementary material for: Multi-drug tolerance in Leishmania persister-like cells
Source: Commun Biol. 2026 Jun 12;9:1263. doi: 10.1038/s42003-026-10456-w (PMC13616925; doi:10.1038/s42003-026-10456-w)
Supplement: Supplementary file 2 [file 42003_2026_10456_MOESM11_ESM.pdf]

nature portfolio

Peer Review File

Multi-drug tolerance in Leishmania persister-like cells

Corresponding Author: Dr Malgorzata Domagalska

This file contains all reviewer reports in order by version, followed by all author rebuttals in order by version.

Version 0:

Reviewer comments:

Reviewer #1

(Remarks to the Author)
In their manuscript entitled “Multi-drug tolerance in Leishmania persister-like cells” Aroni-Soto et al describe the use of ten cloned L. donovani strains with different levels of genomic pre-adaptation to antimony to introduce eGFP into the 18S rDNA locus using a pLEXSY-hyg derived construct to study the interplay between drug resistance and quiescence in parasite survival under potassium antimonium tartrate (PAT) pressure using a strain-specific normally lethal dose. The drug killed the majority of promastigotes, but also led to a small population of quiescent cells. These were identified by a reduction in eGFP expression – a proxy of metabolic activity. Out of four viability profiles, they chose a cell line representing no pre-adaptation to PAT (BPK026) and one with H-locus amplification together with the AQP1 indel associated with antimony resistance (BPK275) for detailed analyses. They tested proliferation and mitochondrial activity using CellTracker, and mitochondrial potential measurements and found that the adoption of a quiescent state was associated with survival. Comparing the genome of controls and post-PAT cells by sequencing could not detect a consistent alteration in parasites analysed from different viability profiles. An underlying mechanism remains to be investigated. PAT-triggered BPK026 quiescent cells gained tolerance to amphotericin B (AMB), miltefosine (MIL), and bortezomib (BOR). The results for BPK275 were more variable, but overall a valid conclusion was drawn as PAT-tolerant cells also show tolerance to other drugs, but the degree of this cross-tolerance is drug- and strain-dependent.
This manuscript provides novel data that will be of significant interest to a wider community. All experiments and statistical analyses have been performed correctly and would allow reproduction. This is a meticulous and very-well performed study, which clearly merits publication in Communications Biology.
General comment:
While overall metabolism is switched into an energy-saving mode in quiescent cells, crucial processes allowing the survival of the cells under stress conditions remain active. What is known in Leishmania about this? GFP expressed from any of these would be a good control to show that it is not anything else that affects GFP fluorescence.

Minor corrections:
Lines 69-75 are not required as the same information is presented in more detail in lines 122-133.

At the beginning of line 349 it would help to provide the expected outcome for each condition to make it easier to understand the results. Something along the lines of: Co-exposure from the beginning means that there is no time to induce quiescence caused by PAT. The % viability ratio goes down because of the additive effect of the drugs. If % viability ratio in serial exposure is lower than in the control it means that there is no effect of PAT-induced quiescence on the second drug. Vice versa, if the % viability ratio stays similar to the control, PAT-induced quiescence also caused resistance to the second drug.

Check IC50 to be with 50 in subscript throughout (including supplement).
Check mL to be capital L throughout (including supplement).
Line 142, …of the rDNA locus…
Line 277, We then, we sequenced
Line 282, changes (Fig. 3A,B; Supp. Fig. 5) 283 changes.
Line 289, and chr 07 in
Line 290, chr 32 and chr
Line 292, and ch 31 in
Line 381m BOR. (Fig. 6B).
Line 411, showed a bi-phasic time-kill
Line 441, not have a different impact

Line 446, a proton
Line 466, studied here exhibited here a
Line 470 suggests
Line 479, persister-like cells in
Line 484, We also unravelled unexpected

Reviewer #2

(Remarks to the Author)
In this manuscript, Aroni-Soto et al. have studied the phenomena of tolerance/persistence in the insect promastigote stage of L. donovani. Persistence is an important phenomenon in the field of chemotherapy, but still ill-defined in Leishmania, thus the importance of this work. The authors have selected a number of isolates with varying susceptibility to trivalent antimony (SbIII) and showed that when in contact with lethal SbIII concentration, a fraction of the cells was in a 'quiescent' mode, although a wide variety of viability profiles were observed. They have attempted also to qualify this quiescence by looking at rRNA expression, cell replication, and mitochondrial potential in cells challenged or not with lethal concentration of SbIII. Sequencing the genomes of various cells who went through quiescence did not reveal any genomic differences. They also observed that strains tolerant to SbIII showed cross-resistance to other drugs but again a variety of profiles were observed.

This was not an easy paper to review. I had to put many more hours than usual. While adding diversity of strains is often of interest, in this case it certainly complicates matters. Yet the authors can be congratulated in trying to assess an issue not well studied in parasitology. Please see my comments below.

1. I have issues with their concept of pre-adaptation. In my expert mind this is not pre-adaptation it is decreased susceptibility (if the authors do not want to use the term resistance) due to either increased copy number of MRPA or SNPs in AQP1. Obviously, cells with decreased susceptibility have all the tools to deal more efficiently with extreme drug pressure. I am wondering when they use BK275 as one of their workhorses for this study whether they really look at quiescence. This will need to be addressed in a fair and square matter.
2. Table 1 should absolutely include EC50s for SbIII or Table 1 and 2 could be combined. Can the profiles listed be in line with the susceptibility values? This is more or less what Table 2 suggests. Why the SNP in AQP1 not included in this Table for strain BPK085? For group P3 and P4 post-SbIII, susceptibility to SbIII has decreased by 2-4 fold. Is this change stable, if cells are grown in absence of drugs? This could help in supporting or excluding quiescence.
3. They use rRNA expression to monitor quiescence. Please specify in the text which rRNA is being looked at. More importantly, what is the evidence that this is a proxy of metabolic activity?
4. Genomic analysis did not reveal changes in P3/P4 post SbIII. They did not detect SNPs common to all replicates. Were SNPs common to at least two replicates? Have the authors excluded that different SNPs could lead to a decreased susceptibility phenotype? Can they confirm that their genomic DNA extraction protocol do not exclude small extrachromosomal DNAs (that could encode MRPA)? Have they looked at intergenic SNPs? A SNP in intergenic regions of MRPA or AQP1 could have consequences on SbIII susceptibility. qTR-PCR of MRPA or AQP1 are feasible experiments in attempts of explaining the increased resistance in the post-SbIII samples shown in Table 2. Transient RNA overexpression would appear as a useful strategy for quiescence. Is Fig 3 helpful? What genes exactly are they comparing??
5. Line 343-344 they state that EC50s for other drugs were comparable between BPK026 and 275. This is not true for paromomycin where 275 is three-fold less susceptible (Table S5). This is likely to explain their data in 6A. The authors may wish to rephrase their sentences at lines 371-372 and 448-449.
6. I find the data with BPK026 when comparing Co vs S the most interesting and a better indication for quiescence. Too bad that they concentrated on BK275 instead of other sensitive strains. The text on cross-tolerance (lines 353-374) is hard to follow and the diversity of phenotypes is hard to reconcile in a unifying model. Please try to clarify.

Reviewer #3

(Remarks to the Author)
Multidrug tolerance in Leishmania persister-like cells.
In this study the authors have shown that there is an evidence of existence of persister like cells in cultured Leishmania donovani induced by treatment with lethal dose of antimony. Most of the cells die but some of the cells survive the crisis and develop into quiescence persister cells. It may be the mechanism of persistence of the disease after recovering from infection. The study is well executed but I have following comments.
Comments:
1. What was the rationale of using potassium antimonium tartrate (PT) to study drug resistance and quiescence since this drug is no longer used for treatment of Leishmaniasis?
2. You are basing alteration in metabolic activity on one component i.e. rEGFP expression. It will be prudent to do unbiased metabolic analyses to observe what all other changes are taking place, which may lead to understanding the pathways that may be altered during the development of quiescent-persister cells.
3. Lines 295-297: Using the methods of sequencing comparing genome of control and post PAT treated cells, you were not able to pinpoint possible signatures of drug resistance except for some minor nucleotide changes.

4. Therefore, it would be important to do transcriptomic analysis, that would identify novel modifications in the quiescence cells and may illustrate the differences between quiescence and non-quiescence cell types.
5. All the experiments described in this manuscript are based on in vitro treatment of Leishmania parasites, it will be important to see if there is evidence of existence of quiescence and persister phenotype in vivo using animal experiments.

Version 1:

Reviewer comments:

Reviewer #1

(Remarks to the Author)
The revised manuscript is much improved and I am happy to see my concerns and the concerns of the other reviewers addressed appropriately. There are only a few minor things listed below the authors might want to consider.
Line 26, Replace “widely” by a more accurate term describing the current use of antimony for treatment.
Line 30, It is "downregulated transcription for core metabolic pathways" as a result, which is observed here, and not the process of "transcriptional downregulation". Also needs to be addressed in line 524.
Ensure consistent spelling of “sand fly”.
Line 551, I assume this should be post-translational modifications.
Line 619, check spelling “transition thorough the quiescent”
Species names in the references should be in italics.

Reviewer #2

(Remarks to the Author)
This is a much improved manuscript at many levels, including in the clarity of the writing that made the reading much more friendly. The inclusion of RNA seq data was helpful in general terms. Overall the authors have adequately dealt with my queries (and queries of others). This study will be of interest to drug aficionados.

Reviewer #3

(Remarks to the Author)
The authors have satisfactorily responded to my comments

Open Access This Peer Review File is licensed under a Creative Commons Attribution 4.0 International License, which permits use, sharing, adaptation, distribution and reproduction in any medium or format, as long as you give appropriate credit to the original author(s) and the source, provide a link to the Creative Commons license, and indicate if changes were made.
In cases where reviewers are anonymous, credit should be given to 'Anonymous Referee' and the source.
The images or other third party material in this Peer Review File are included in the article’s Creative Commons license, unless indicated otherwise in a credit line to the material. If material is not included in the article’s Creative Commons

license and your intended use is not permitted by statutory regulation or exceeds the permitted use, you will need to obtain permission directly from the copyright holder.
To view a copy of this license, visit https://creativecommons.org/licenses/by/4.0/

Manuscript COMMSBIO-25-2039-T

Rebuttal letter: our responses to reviewers' comments are in blue; when needed, we copied (in italics) the text that was modified in the manuscript and indicate the corresponding lines (in the clean version of the manuscript).

Comments from editors

For all graphs depicting a single point value (e.g., mean) with error bars, you must add individual data points or convert the graph to a boxplot or dot-plot to show data distribution. [Done]

It's mandatory to provide access to the numerical source data for graphs and charts either through a repository or by providing the data in a Supplementary Data file (in Excel format). [Done]

All blots/gels must be accompanied by size markers in every figure panel. Uncropped and unedited blot/gel images must be included as Supplementary Figure(s) in the Supplementary Information pdf. Not applicable

Please ensure that you have complied with the data deposition policies at the Nature Portfolio; please see here. [Done]

Please ensure that you have complied with our policies on research involving animals and humans, see here Not applicable

Please follow the ARRIVE guidelines for reporting animal experiments. Please fully complete an ARRIVE checklist including both the essential and recommended set of items (adding information to the manuscript where needed) and upload this with your revised manuscript. Not applicable

Please also see our revision checklist for guidance on formatting the manuscript and complying with our policies. A comprehensive guide to our formatting requirements for final submissions is also available for your reference here. See attached revision and editorial checklists

II. Comments from reviewers

Reviewer #1:

1. General comment:
While overall metabolism is switched into an energy-saving mode in quiescent cells, crucial processes allowing the survival of the cells under stress conditions remain active. What is known in Leishmania about this? GFP expressed from any of these would be a good control to show that it is not anything else that affects GFP fluorescence.

> Response: In our previous work we demonstrated that rEGFP expression is a suitable tool for monitoring quiescence as a negative biomarker: see also response to comment 3 of reviewer 2. In addition, the rEGFP results were accompanied by

replication analysis (Cell tracker), mitochondrial membrane potential and transcriptomic analysis of the two selected strains (BPK026 and BPK275), which was added in the revised version of the manuscript. Transcriptomic analyses of PAT-exposed quiescent BPK026 and BPK275 allowed us to identify molecular pathway modified in quiescent PAT-exposed *L. donovani.* For details, see also response to comment 4 of reviewer 3.

2. Minor corrections:
Lines 69-75 are not required as the same information is presented in more detail in lines 122-133.
> Response: indeed, the corresponding text was deleted

At the beginning of line 349 it would help to provide the expected outcome for each condition to make it easier to understand the results. Something along the lines of: Co-exposure from the beginning means that there is no time to induce quiescence caused by PAT. The % viability ratio goes down because of the additive effect of the drugs. If % viability ratio in serial exposure is lower than in the control it means that there is no effect of PAT-induced quiescence on the second drug. Vice versa, if the % viability ratio stays similar to the control, PAT-induced quiescence also caused resistance to the second drug.
> Response: we tried to make the text easier to follow, by inspiring us from the comments of the reviewer. See lines 383-391.
"…To assess the protective effect of PAT-associated tolerance, we first triggered quiescence by exposing the promastigotes to PAT (strain-specific normally lethal doses) for 5 days, followed by 5 days of simultaneous exposure of PAT and the additional drug (serial exposure (S), Supp. Table 7). ***If the viability ratio for a given drug was similar to that of PAT, it would mean -in serial exposure- that PAT-induced quiescence provides cross-tolerance to the second drug.*** *Secondly, we added both PAT and the second drug from the very beginning (combination exposure (Co), Supp. Table 7).* ***In that case, a decrease of the viability index of the combination would be due to the additive effect of the drugs (in that experimental scenario, there is no time to induce quiescence with PAT)****…"*

Check IC50 to be with 50 in subscript throughout (including supplement). [done]
Check mL to be capital L throughout (including supplement). [done]
Line 142, …of the rDNA locus… [done]
Line 277, We then, we sequenced [done]
Line 282, changes (Fig. 3A,B; Supp. Fig. 5) 283 changes. [done]
Line 289, and chr 07 in [done]
Line 290, chr 32 and chr [done]
Line 292, and ch 31 in [done]
Line 381m BOR. (Fig. 6B). [done]
Line 411, showed a bi-phasic time-kill [done]
Line 441, not have a different impact [done]
Line 446, a proton [proton-dependent transport processes]
Line 466, studied here exhibited here a [The 11 *L. donovani* lines studied here exhibited a range of quiescence and persister phenotypes]
Line 470 suggests [done]

Line 479, persister-like cells in [?]
Line 484, We also unravelled unexpected [done]

Reviewer #2:

1. I have issues with their concept of pre-adaptation. In my expert mind this is not pre-adaptation it is decreased susceptibility (if the authors do not want to use the term resistance) due to either increased copy number of MRPA or SNPs in AQP1. Obviously, cells with decreased susceptibility have all the tools to deal more efficiently with extreme drug pressure.
> Response: albeit discussable (See our 2018 paper: Molecular Preadaptation to Antimony Resistance in *Leishmania donovani* on the Indian Subcontinent. Dumetz et al. mSphere. 2018 Apr 18;3(2):e00548-17. doi: 10.1128/mSphere.00548-17), we decided to follow the recommendation of the referee.
See lines 92-98: "*ii) whether the presence of **genomically defined resistance** to antimony has an impact on the quiescence and persister phenotypes under PAT exposure. We found compelling evidence that quiescence is a universal survival mechanism, characterised by a common transcriptional signature adopted by genomically distinct L. donovani strains. However, the impact of the transition through quiescence, on drug susceptibility differed, depending on the presence of **genomically determined antimony resistance**. In drug susceptible parasites, the antimony tolerance phenotype was reversible in non-drug conditions.*"

See lines 114-121. "*We selected ten cloned strains of L. donovani isolated in the ISC 16, with different levels of susceptibility to antimony: i) three strains where **no obvious genomic changes indicative of PAT resistanc**e are known to be present (ISC1); these strains are highly susceptible to PAT; ii) seven strains belonging to different genetic groups (ISC4, 6, and 9) characterized by possessing **one known genomic marker of resistance to PAT**, the intra-chromosomal amplification of the H-locus, which contains the MRPA gene, a **known driver of antimony resistance**; these strains show decreased susceptibility to PAT; iii) one strain with low susceptibility has **two known genomic drivers of PAT-resistance**: a 2-nt insertion (458_459dupTC|p.Ala154fs) that introduces a stop codon in the aqp1 gene [16…]*"

See lines 155-157: "…*BPK275, a strain with **two genomic signatures of resistance** to antimony was the least affected by PAT having a viability levels remaining above 69% for the entire exposure asssay (Supp. Table 2).*"

See lines 174-177: "…*To confirm the quiescent status of the viable cells under PAT, we selected two lines, BPK026 and BPK275, which present the most extreme viability profiles (P1, and P4, respectively) and **extreme genomic signatures of susceptibility to PAT** (no amplification of H locus and wild type AQP1 for BPK026 and amplification of H locus and truncated AQP1 for BPK275) for more in-depth characterization.*

See lines 359-362: " *Overall, the impact of the transition through quiescence, on drug susceptibility differed, depending on the presence of **pre-existing antimony resistance**. Lines without any **signature of PAT resistance** exhibited features of a classical quiescence phenomenon linked to the presence of persisters, where increased tolerance to a drug is restricted to the quiescence phase…*"

See lines 435-437: *"…Quiescence was detected in all parasites with **pre-existing genomic signatures of PAT resistance**, and these cells could also resume growth while still under treatment exposure, enhancing the level of resistance to PAT in so doing"*

See lines 528-529: "…*We also unravelled unexpected effects of adoption of quiescence, which may go beyond the period of exposure to drug. In strains with **pre-existing drug resistance,**"*

I am wondering when they use BK275 as one of their workhorses for this study whether they really look at quiescence. This will need to be addressed in a fair and square matter.
> Response: our paper was indeed focusing on quiescence, but the inclusion of a highly resistant strain like BPK275 allowed us to study the interplay between drug resistance and quiescence. This was already mentioned in the previous version of the text.
See lines129-131: *"…By using this collection of parasite isolates from the field with different levels of susceptibility to antimony we aimed to study the potential interplay between different heritable, genetically defined forms of drug resistance and quiescence in parasite survival under PAT pressure"*

2. Table 1 should absolutely include EC50s for SbIII or Table 1 and 2 could be combined. Can the profiles listed be in line with the susceptibility values? This is more or less what Table 2 suggests. Why the SNP in AQP1 not included in this Table for strain BPK085?
> Response: Indeed, this was adjusted and Tables 1 and 2 were merged, see now the new Table 1 at the end of the manuscript, lines 1010-1019 and here below in the rebuttal letter. In the table, we only mentioned the indel (458_459dupTC|p.Ala154fs) that was causing a stop codon in all 52 isolates of ISC5 *L.donovani* (mentioned at the bottom of table 1, lines 1017-1018). The truncated frameshift protein found in ISC5 was predicted to be incapable of forming a functional trans-membrane channel (Imamura et al., 2016). The SNP in AQP1 of BPK085 is present at a different location (94_95dupAA|p.Asn32fs) and was only encountered in one isolate.

This is mentioned in the text at lines 120-126
"…iii) one strain with low susceptibility has two known genomic drivers of PAT-resistance: a 2-nt insertion (458_459dupTC|p.Ala154fs) that introduces a stop codon in the aqp1 gene [16], proposed to be associated with antimonial resistance observed in the ISC5 genotype in addition to the intra-chromosomal amplification of the H-locus; the strain shows a low susceptibility to PAT [16,30]. We also included a reference L. donovani strain isolated in East Africa (LdBob), highly susceptible to antimony (Table 1) [34,35]. Of note, one of the strains belonging to ISC6 group, BPK085 also carries a different 2-nt insertion mutation (94_95dupAA|p.Asn32fs) in the aqp1 gene, which results in a frameshift [16]. …"

*"…**Table 1.** Cloned strains of Leishmania donovani isolates from the Indian Subcontinent and reference strain (LdBOB) with their respective drug susceptibility in promastigotes (IC50, µM). The values were measured by resazurin assay after removing normally lethal PAT dose 10-day (post-PAT); and the control (No PAT) was*

maintained in parallel and not exposed to PAT. For three lines that did not recover after drug exposure, the data is not reported and denoted as 'n.d.' (not determined). The data is presented as the mean ± SEM of three biological and technical replicates.

Strain	ISC population	H-locus amplified	AQP1 indel	IC_{50} PAT No PAT	IC_{50} PAT Post PAT
**Profile 1***					
BPK026	ISC1	no	no	9.0 ± 0.6	6.7 ± 0.7
BPK031	ISC1	no	no	19.0 ± 1.2	20.9 ± 4.6
BPK156	ISC1	no	no	18.2 ± 0.7	19.7 ± 0.1
**Profile 2***					
BPK190	ISC4	yes	no	nd	nd
BPK087	ISC4	yes	no	nd	nd
LdBOB	Other	no	no	nd	nd
**Profile 3***					
BPK085	ISC6	yes	yes[@]	89.9 ± 0.5	241.5 ± 14.7
BPK282	ISC6	yes	no	137.1 ± 11.9	423.5 ± 2.8
BPK294	ISC9	yes	no	40.2 ± 4.4	230.1 ± 13.9
**Profile 4***					
BPK080	ISC4	yes	no	52 ± 1.7	86.8 ± 2.6
BPK275	ISC5	yes	yes[†]	46.4 ± 0.5	214.1 ± 30.1

** Profiles 1-4 correspond to patterns of survival under normal lethal PAT dose.*
[†] 2-nt Indel in position 458_459dupTC|p.Ala154fs of the AQP1 locus, causing a stop-codon, present in all ISC5 isolates[16].
[@] 2-nt indel in position 94_95dupAA|p.Asn32fs of the AQP1 locus…"

For group P3 and P4 post-SbIII, susceptibility to SbIII has decreased by 2-4 fold. Is this change stable, if cells are grown in absence of drugs? This could help in supporting or excluding quiescence.

> Response: The experimental setup to measure SbIII susceptibility in the parasites recovered from drug-exposure was the following:
The post-SbIII strains were transferred to M199-supplemented medium without drug pressure and maintained until recovery was microscopically confirmed by the presence of motile promastigotes, the recovery duration varied from 0 days (BPK275, which was already proliferating) to 5 weeks (BPK026). Once microscopically visible parasite growth was observed, the parasites had one passage in M199-supplemented medium for 3 days (showing normal parasite growth) before IC_{50} measurement.

We did not systematically check the stability of this hyper-resistance and we believe this is out of the scope of this manuscript. In a pilot experiment with BPK294, we compared the post-SbIII IC_{50} after 1 passage and 10 passages in culture medium without drug. Result was 183 and 196 µM, respectively, suggesting that the increased resistance phenotype is stable.

This was mentioned now in the M&M. See lines 637-643: *"We measured the PAT susceptibility of the cells that recovered from the PAT exposure by comparing the IC50 without drug exposure (No PAT) and after removal of PAT and recovery of growth (Post-PAT). The post-PAT strains were transferred to M199-supplemented medium without drug pressure and maintained until recovery was microscopically confirmed by the presence of motile promastigotes. The recovery duration varied from 0 days (BPK275, which was already proliferating) to 5 weeks (BPK026). Once microscopically visible parasite growth was observed, the parasites had one passage in M199-supplemented medium for 3 days (showing normal parasite growth) before IC50 measuring."*

3. They use rRNA expression to monitor quiescence. Please specify in the text which rRNA is being looked at. More importantly, what is the evidence that this is a proxy of metabolic activity?
> Response:
With our rEGFP biosensor, we look at the expression of the 18S ribosomal DNA locus. This was already mentioned in the M&M section of the text. For each strain, the enhanced green fluorescent protein (EGFP) was integrated within the 18S ribosomal DNA locus (rEGFP) of promastigotes, with pLEXSY-hyg system (Jena Bioscience).

We published several papers showing that the expression of rEGFP constituted a proxy of metabolic activity and a good biosensor to monitor the quiescence cycle. In Jara et al. (2017), we described during quiescence a drop in the levels of macromolecules involved in the ribosome biogenesis (ribosomal RNA and proteins), a signature of quiescence encountered from prokaryotes as Mycobacterium to eukaryotes as Saccharomyces. Based on these findings, we developed a bio-sensor of quiescence based on integration of GFP within the 18S rDNA locus (Jara et al., 2019). We hypothesized that the expression of this reporter gene driven by the ribosomal promoter may serve as a biosensor of the metabolic activity of the parasite. We showed that (i) rGFP expression decreased significantly and rapidly during the in vitro transition from extracellular promastigotes to intracellular amastigotes, (ii) the decrease in rGFP expression was coupled in vitro with a decrease in replication as measured by BrdU incorporation, (iii) modulation of rGFP

expression both in vitro and in vivo was reversible, the signal of the reporter gene increasing again during the transition from amastigote to promastigote. Last but not least, the decrease in rEGFP expression during quiescence agreed with the transcriptional shift and metabolomic down-regulation (Jara et al., 2022).

4. Genomic analysis did not reveal changes in P3/P4 post SbIII. They did not detect SNPs common to all replicates. Were SNPs common to at least two replicates? Have the authors excluded that different SNPs could lead to a decreased susceptibility phenotype? Can they confirm that their genomic DNA extraction protocol do not exclude small extrachromosomal DNAs (that could encode MRPA)? Have they looked at intergenic SNPs?
> Response: we followed the suggestions of the reviewer and added the following in text (lines 331-339)
"Because of ISC1 strains/P1 parasites having much higher number of heterozygous SNPs in comparison to other strains 16, we carried out the analysis of P1 and P3/4 parasites separately; but we searched SNPs present in three replicates of post SbIII and not detected in non-drugged controls (Post-PAT). In both P1 and P3/4 lines, we observed 56 SNPs but did not observe consistent (i.e. present in all three replicates) SNP/indel changes (Supp. Fig. 6), including in intergenic regions of AQP1 and MRPA. The analysis was repeated with lower stringency, considering SNPs common to at least two replicates (and not detected in controls). This revealed more hits (321), but we found no variants (linked to the same locus), which were specific to P3/P4 parasites, and as such could underlie the observed hyper-resistance (see supplement and Data 7)."

> We have not checked for the presence of extrachromosomal DNA, but we performed transcriptomic analysis to identify potential transcriptomic adaptations of POST PAT BPK275 parasites that could explain their hyper-resistant phenotype. We observed that the replicative parasites recovered from PAT (POST-PAT) exposure have almost identical transcriptional profile as the no PAT counterpart as evidenced by extremely low number of DE genes. No changes in MRPA or other ABC transporters could be detected in hyper-resistant POST-PAT BPK275, thus the presence of extrachromosomal DNA carrying MRPA or other drug-resistance genes is unlikely.

A SNP in intergenic regions of MRPA or AQP1 could have consequences on SbIII susceptibility.
> Response: we checked this. For AQP1, 2 upstream variants were detected, only in ISC1 strains BPK026, BPK031 and BPK156 and in three replicates; they were found in post SbIII strains and respective control. For MRPA, 11 variants (upstream and downstream) were detected in the same ISC1 strains and in three replicates. Like for AQP1, they were found in post SbIII strains and respective control. Accordingly, the changes in SbIII susceptibility occurring during the experiment were not due to intergenic SNPs.

See lines 334-339. *"In both P1 and P3/4 lines, we observed 56 SNPs but did not observe consistent (i.e. present in all three replicates) SNP/indel changes (Supp. Fig. 6), including in intergenic regions of AQP1 and MRPA. The analysis was repeated with lower stringency, considering SNPs common to at least two replicates (and not detected in controls). This revealed more hits (321), but we found no variants (linked*

to the same locus), which were specific to P3/P4 parasites, and as such could underlie the observed hyper-resitance (see supplement and Data 7)."

qTR-PCR of MRPA or AQP1 are feasible experiments in attempts of explaining the increased resistance in the post-SbIII samples shown in Table 2. Transient RNA overexpression would appear as a useful strategy for quiescence.
> Response: Instead of qRT-PCR, we chose to use transcriptomic analyses to answer this question. We investigated the expression of MRPA and the related ABC transporters in the non-exposed (parasites before- and Post-PAT exposure, and in drug-exposed cells at D2 in BPK275 and BPK026 as a control (line with unchanged IC50 before- and Post-PAT exposure). We found the expression of several ABC transporters to be transiently upregulated in BPK275 at D2, but there were no clear differences in their expression when No PAT (before drug exposure) parasites were compared to POST PAT BPK275.
We did not analyse in detail the expression of AQP1 as we know that it is not functional in BPK275 and therefore the changes in its expression should not make a further impact on the hyper-resistance phenotype.

See lines results section lines 308-327: *"As the main genomic difference between the P1 and P3/P4 lines is the increased copy of the MRPA, a membrane ABC transporter known to be involved in resistance to antimony, we investigated whether increased levels of MRPA could underlie the observed reduction in PAT susceptibility in Post-PAT P3/P4 parasites. To address this, we made use of transcriptomic data generated for one P1 and P4 line (BP026 and BPK275, respectively) described above. We plotted Reads Per Kilobase Million (RPKM) values for both strains in replicative parasites before- and Post-PAT exposure, and in drug-exposed cells at D2 (both lines), and investigated MRPA transcript levels. As expected, we found MRPA transcript to be more abundant (2.64 to 2.76) in BPK275, but no further changes were observed during and post-PAT exposure (Supp. Fig 5). Next, we decided to assess the expression of other members of the ABC protein family, which were initially described in L. major and of which some were shown to be linked to drug resistance [41,42]. We found that 13 other ABC transporters showed higher transcript abundance in BPK275 both with and without drug exposure, but no specific increase could be found in BPK275 parasites that transitioned through quiescence. Further inspection revealed a subset of 12 ABC transporters that showed higher abundance in BPK275 as compared to BPK026 specifically during the quiescence period (D2), further supporting potentially higher ability to remove antimony in BPK275 cells exposed to drug, even during the quiescence period (Supp. Fig 5). Lack of clear increase in transcript levels of the ABC transporters in PostPAT BPK275, together with negligible transcriptional differences between BPK275 no PAT and post PAT (4 DE genes, ($|log2FC| \geq 1$, adjpvalue>0.05) suggests that phenomena other than transcriptional adaptations are responsible for the increase in IC50 after the transition through drug-triggered quiescence in the P3/P4 parasites…"*

See also discussion, lines 474-481 ; "…*we demonstrated that neither general transcriptional remodelling nor an increase in the transcript levels of MRPA or related ABC transporters underlie this hyper-resistant phenotype. As the gene encoding the MRPA pump which removes antimony from the cells 51 is already amplified as part of the H-locus in resistant cells, we cannot exclude its levels are further increased through post-transcriptional modifications in these strains. Translational*

reprogramming previously reported as a mechanism of antimony resistance 52 could also drive the observed hyper-resistant phenotype. It will be interesting to explore this in future work..".

In addition, we observed that the replicative parasites recovered from PAT (POST-PAT) exposure have almost identical transcriptional profile as the no PAT counterpart as evidenced by extremely low number of DE genes. This suggests that the observed hyper-resistance in BPK275 line is through other than transcriptional adaptations.

See lines 278-284: *"Collectively, these transcriptomic results confirm strong metabolic downregulation accompanied by transcriptional reprogramming in both strains exposed to PAT, which is consistent with the adoption of a quiescent state. Accordingly, this transcriptional adaptation is transient and replicative parasites recovered from PAT (POST-PAT) exposure have an almost identical transcriptional profile as their no PAT counterparts as evidenced by an extremely low number of DE genes (4 and 33, (|log2FC|□1, adjpvalue>0.05 for BPK275 and BPK026, respectively, Supp. Table 4)."*

Is Fig 3 helpful? What genes exactly are they comparing??
> Response: Indeed, this figure is not useful and was deleted from the revised ms. In the Supp. Fig. 6, the same information is mentioned in individual heatmaps, one per strain. This supp. Figure is complemented by the Data set 7in which SNPs present in 2/3 replicates of P3/P4 post SbIII are mentioned, see above.

5. Line 343-344 they state that EC50s for other drugs were comparable between BPK026 and 275. This is not true for paromomycin where 275 is three-fold less susceptible (Table S5). This is likely to explain their data in 6A. The authors may wish to rephrase their sentences at lines 371-372 and 448-449.
> Response: indeed, this was modified in the text

See lines 381-383: *"First, we found that the IC50 values for each of these compounds were comparable in BPK026 and BPK275 (Supp. Table 6), except for PARO to which BPK275 was three-fold less susceptible ."*

See also lines 412-415: "*…for PARO a higher viability ratio in Co exposure in comparison to C and a ratio close to the control under serial exposure, suggesting that PARO and PAT act synergistically to trigger the presence of quiescent cells, but also a result that could be partly explained by the low susceptibility of BPK275 to PARO.…"*

See also lines 497-500: "…As an inhibitor of protein synthesis paromomycin likely continues to act on processes crucial to the viability of quiescent cells, which would explain the absence of cross-tolerance to this drug. However, this last result could be biased by the intrinsic low susceptibility of BPK275 to paromomycin…"

6. I find the data with BPK026 when comparing Co vs S the most interesting and a better indication for quiescence. Too bad that they concentrated on BK275 instead of other sensitive strains.
> Response: As mentioned above, we aim here to study the interplay between drug resistance and quiescence triggered drug tolerance.

The text on cross-tolerance (lines 353-374) is hard to follow and the diversity of phenotypes is hard to reconcile in a unifying model. Please try to clarify.
> Response: we tried to make this text easier to follow; see our response to comment of reviewer 1. The diversity of phenotypes illustrates the variety of adaptive strategies of Leishmania and the key conclusion about cross-tolerance is that "*Collectively, these results show that PAT-tolerant cells also show tolerance to other drugs, but the degree of this cross-tolerance is drug- and strain-dependent..*" (see lines 416-417)

Reviewer #3:

1. What was the rationale of using potassium antimonium tartrate (PT) to study drug resistance and quiescence since this drug is no longer used for treatment of Leishmaniasis?
> Response: Antimonials are indeed not used anymore in the Indian sub-continent, but they are still used in Africa and Latin America (not only against visceral leishmaniasis). Most knowledge on drug resistance and tolerance was acquired through clinical or experimental studies with PT; this also includes our previously published papers (51 papers, source Pubmed). Present study can benefit from all this previously accumulated knowledge. Furthermore, populations of *L. donovani* have evolved for decades through human-to-human transmission under antimony pressure (Imamura et al., 2016); this likely had an impact on molecular adaptations to antimony. Last but not least, the 10 strains of the Indian subcontinent here studied were isolated in a clinical of antimonial therapy. Thus, using PT in present study is coherent with clinical and experimental contexts.

2. You are basing alteration in metabolic activity on one component i.e. rEGFP expression. It will be prudent to do unbiased metabolic analyses to observe what all other changes are taking place, which may lead to understanding the pathways that may be altered during the development of quiescent-persister cells.
> Response: indeed, but this is not the scope of the paper. As mentioned in the answer to reviewer1's comment, in our previous work (Jara et al., 2022), we showed that quiescence in *Leishmania lainsoni* was indeed accompanied by (i) reduction in overall transcription, but a subset of transcripts did not follow this trend and were relatively more abundant in quiescent populations cells (amastins, GP63 or processes like autophagy) and (ii) the metabolome followed a similar trend of overall downregulation, accompanied by upregulation of some metabolites like those involved in carbon sources as an alternative to glucose. In the meantime, the best tool available in our hands if a negative biomarkers, i.e. the expression of rEGFP. In addition, we performed transcriptomic analyses to characterize molecular adaptations in these quiescent *L. donovani* cells. See new sections, (i) results: lines 220-284; (ii) discussion: 451-466 and (iii) M&M: 670-716 (not copied here)

3. Lines 295-297: Using the methods of sequencing comparing genome of control and post PAT treated cells, you were not able to pinpoint possible signatures of drug resistance except for some minor nucleotide changes.
> Response: see above our response to comment 4 of reviewer 2.

4. Therefore, it would be important to do transcriptomic analysis, that would identify novel modifications in the quiescence cells and may illustrate the differences between quiescence and non-quiescence cell types.

> Response: To address this comment, we performed transcriptomic analyses of BPK026 and BPK275 that allowed us to identify molecular pathway modified in quiescent PAT-exposed *L. donovani.* The results of these new analyses are added in the revised version of the manuscript. Briefly, we identified several core metabolic pathways to be downregulated in both strains, which is consistent with the rEGFP data, reduced mitochondrial potential and reduced growth and proliferation. In addition, we identified autophagy and membrane trafficking as well as several genes, including amastins and amastin-like proteins to be upregulated in the quiescent cells of both strains.
See new sections, (i) results: lines 220-284; (ii) discussion: 451-466 and (iii) M&M: 670-716 (not copied here)

5. All the experiments described in this manuscript are based on in vitro treatment of Leishmania parasites, it will be important to see if there is evidence of existence of quiescence and persister phenotype in vivo using animal experiments.

> Response: as written in the introduction (lines 72-77), "…*Quiescence in Leishmania was inferred in mice when amastigotes of L. mexicana were shown to have a very low replication rate, together with low rates of RNA synthesis, protein turnover, and membrane lipid synthesis* [26]*. Another mouse study revealed heterogeneity among L. major amastigotes with non-replicating parasites distinguished from slowly replicating populations* [27]*. Another study of L. mexicana in mice revealed quiescent populations within collagen-rich mesothelium surrounding lesions, where miltefosine accumulation was also limited* [24]*.*"

We are conscious that our observations would need to be verified in an intra-cellular context, both in vitro and in vivo. Present paper represents a first step in this direction, using models compatible with high-throughput analyses.
At the very end of the paper, we added a corresponding statement, see lines 532-533; "…*Further work is required to verify our observations in an intra-macrophage context, both in vitro and in vivo…"*

Revision of ms COMMSBIO-25-2039A (V140426), numbering as on version with track change (all mark-up!)

1. Comments of referee 1

Line 26, Replace “widely” by a more accurate term describing the current use of antimony for treatment. Changed to ‘still’

Line 30, It is "downregulated transcription for core metabolic pathways" as a result, which is observed here, and not the process of "transcriptional downregulation". Also needs to be addressed in line 524. Done in lines 30-31 and 457-8.

Ensure consistent spelling of “sand fly”. Verified

Line 551, I assume this should be post-translational modifications. Done in line 486

Line 619, check spelling “transition thorough the quiescent” Changed to ‘through’ in line 536

Species names in the references should be in italics. Done in the clean version

2. Shortening of the text

As requested by the editor, we have significantly shortened the text of the ms, from 7145 words to 5351. This done -with justification in comments- by

- Moving parts of the text to Supplement or the legend of figures (highlighted in yellow)
- Editing/shortening some sections (highlighted in green)
- Deleting some sections (highlighted in blue)

Other changes in the text are marked by track change
