# Supplementary material for: Multi-drug tolerance in Leishmania persister-like cells
Source: Commun Biol. 2026 Jun 12;9:1263. doi: 10.1038/s42003-026-10456-w (PMC13616925; doi:10.1038/s42003-026-10456-w)
Supplement: Supplementary file 3 [file 42003_2026_10456_MOESM1_ESM.pdf]

(Aroni-Soto_supp.pdf) Supplementary Information for the ms

Multi-drug tolerance in *Leishmania* persister-like cells

Allison Aroni-Soto[1,2,5], Pieter Monsieurs[1,5], Odin Goovaerts[3], Kaoutar Choukri[1,5], Basudha Khanal[4], Wim Adriaensen[3], Jean-Claude Dujardin[2, 5], Michael P. Barrett[6], Malgorzata A. Domagalska[1,5*]

[1] Institute of Tropical Medicine, Experimental Parasitology Unit, Antwerp, Belgium

[2] Department of Biomedical Sciences, University of Antwerp, Belgium

[3] Institute of Tropical Medicine, Clinical Immunology Unit, Antwerp, Belgium

[4] Department of Microbiology, BP Koirala Institute of Health Sciences, Dharan, Nepal

[5] Institute of Tropical Medicine, Molecular Parasitology Unit, Antwerp, Belgium

[6] School of Infection & Immunity, College of Medical, Veterinary and Life Sciences, University of Glasgow, Glasgow, UK

*__Correspondence:__

Malgorzata A. Domagalska: mdomagalska@itg.be

Content

Supplementary tables and datasets p.2-9

Supplementary figures p.10-21

Supplementary text

- Transcriptional analyses p.22
- Genomic analyses p.22-23
- Models for further studies p.23

Bibliography p.24

SUPPLEMENTARY TABLES & Data sets

Supplementary Table 1. Lowest PAT concentration at which no metabolic activity could be detected among promastigotes (see also Supp. Fig. 1), also called normally lethal dose.

**Strain**	**PAT μM**
**P1**	
BPK026	50
BPK031	100
BPK156	200
**P2**	
BPK190	400
BPK087	400
LdBOB	400
**P3**	
BPK085	400
BPK282	600
BPK294	400
**P4**	
BPK080	400
BPK275	200

Supplementary Table 2. Evolution of the viability percentage of promastigotes during 10 days of exposure to strain-specific normally lethal PAT doses. The values are represented as the mean of 3 biological replicates ± SEM.

Strain	Viability (%)							
	**D0**	**D1**	**D2**	**D3**	**D4**	**D6**	**D8**	**D10**
**P1**								
BPK026	100 ± 0	94.8 ± 0.1	73.2 ± 2.4	25.1 ± 1.6	5.1 ± 0.5	0.7 ± 0.2	0.5 ± 0.4	0.5 ± 0.2
BPK031	100 ± 0	92 ± 1.6	44.6 ± 1.2	11.1 ± 1	5 ± 0.3	1.3 ± 0.5	0.6 ± 0.3	0.5 ± 0.1
BPK156	100 ± 0	69.5 ± 5.9	51.4 ± 9.8	43.6 ± 7.4	22.7 ± 2.8	3.4 ± 1.3	0.6 ± 0.1	0.1 ± 0.1
**P2**								
BPK190	100 ± 0	58.2 ± 5.9	6 ± 1.6	1.4 ± 0.9	0.8 ± 0.5	0 ± 0	0 ± 0	0.5 ± 0.1
BPK087	100 ± 0	57 ± 4.5	11 ± 0.6	2.4 ± 0.3	0.6 ± 0.2	0.1 ± 0.1	0 ± 0	0.5 ± 0.3
LdBOB	100 ± 0	22.9 ± 0.2	1.8 ± 0.3	5.5 ± 0.9	0.1 ± 0.1	0 ± 0	0 ± 0	0 ± 0
**P3**								
BPK085	100 ± 0	84.9 ± 2.6	35.9 ± 6.6	6.8 ± 1	1.9 ± 0.2	0.4 ± 0.3	0.8 ± 0.4	5.5 ± 3.3
BPK282	100 ± 0	60 ± 0.4	15 ± 1.5	4.5 ± 0.9	3.2 ± 0.3	0.7 ± 0.2	0.9 ± 0.4	14.9 ± 4.5
BPK294	100 ± 0	82.8 ± 2.4	39.1 ± 6.8	0.4 ± 0.2	3.4 ± 0.8	0.2 ± 0.1	0.1 ± 0.1	3.1 ± 0.6
**P4**								
BPK080	100 ± 0	85.3 ± 1.9	68.5 ± 6.2	54.4 ± 11.3	36.8 ± 14.1	10.2 ± 3.2	20.2 ± 7.4	53.1 ± 8.6
BPK275	100 ± 0	92.1 ± 0.8	83 ± 0.9	75.7 ± 0.9	69.7 ± 1.4	80.1 ± 2.3	82.2 ± 1.5	92.3 ± 0.5

Supplementary Table 3. Reactivation of parasite proliferation (numbers out of 3 replicates) after PAT exposure for 4 and 10 days, at strain-specific normally lethal dose.

Strain	H-locus amplified	AQP1 indel†	Reactivate after 4 day	Reactivate after 10 day
**P1**				
BPK026	no	no	3 of 3	3 of 3
BPK031	no	no	3 of 3	3 of 3
BPK156	no	no	3 of 3	3 of 3
**P2**				
BPK190	yes	no	3 of 3	0 of 3
BPK087	yes	no	3 of 3	1 of 3
LdBOB	no	no	3 of 3	0 of 3
**P3**				
BPK085	yes	no	3 of 3	3 of 3
BPK282	yes	no	3 of 3	3 of 3
BPK294	yes	no	3 of 3	3 of 3
**P4**				
BPK080	yes	no	3 of 3	3 of 3
BPK275	yes	yes	3 of 3	3 of 3

† Indel associated with antimonial resistance.

Supplementary Table 4. Summary of differentially expressed genes identified under log2 fold-change cutoff 1, using a fixed false discovery rate (FDR) threshold of 0.05 for all comparisons. Breakdown of upregulated and downregulated genes, including the number and proportion of genes classified as hypothetical ("hypo").

**Comparison**	**N_up**	**N_up_hypo**	**%up_hypo**	**N_down**	**N_down_hypo**	**%_down_hypo**
BPK026_PAT_D2_vs_NoPAT	1000	465	46	1592	470	30
BPK275_PAT_D2_vs_NoPAT	206	82	40	241	58	24
BPK026_Post_PAT_vs_NoPAT	18	0	0	15	1	7
BPK275_Post_PAT_vs_NoPAT	4	1	25	0	0	0
NoPAT_BPK275_vs_BPK026	429	137	32	192	62	32
PAT_D2_BPK275_vs_BPK026	1544	494	32	788	343	44
Post_PAT_BPK275_vs_BPK026	421	125	30	232	87	38

Supplementary Table 5. Copy values H-locus (per haploid genome) of each *L. donovani* line after removal of PAT exposure (Post PAT), and controls without exposure (No PAT). The table shows the mean of three biological replicates ± SEM.

Strain	H-locus amplified	H-locus copy values No PAT	H-locus copy values Post PAT
**P1**			
BPK026	no	0.88 ± 0.01	0.93 ± 0.03
BPK031	no	0.93 ± 0.01	0.90 ± 0.01
BPK156	no	0.95 ± 0.01	0.94 ± 0.01
**P3**			
BPK085	yes	2.73 ± 0.03	2.78 ± 0.03
BPK282	yes	2.75 ± 0.003	2.82 ± 0.09
BPK294	yes	1.81 ± 0.04	1.84 ± 0.02
**P4**			
BPK080	yes	1.91 ± 0.01	1.83 ± 0.03
BPK275	yes	2.82 ± 0.03	2.69 ± 0.02

Supplementary Table 6. Susceptibility (IC_{50}) of BPK026 and BPK275 promastigotes to drugs with different modes of action: (i) used to treat leishmaniasis (MIL, Miltefosine; AmB, Amphotericin B, PARO, Paromomycin and (ii) in development (BOR, Bortezomib and FCCP (Carbonycil). Each value represents the mean of three biological replicates ± SEM.

Strain	MIL IC50 µM	AMB IC50 nM	PARO IC50 µM	FCCP IC50 µM	BOR IC50 nM
**P1**					
BPK026	17.8 ± 0.1	64.2 ± 0.5	8.7 ± 0.1	20.0 ± 0.3	54.5 ± 0.6
**P4**					
BPK275	13.0 ± 0.1	58.7 ± 0.7	21.4 ± 1.4	14.6 ± 0.5	55.5 ± 5.1

Supplementary Table 7. Experimental design for the study of multidrug tolerance, first exposure is made for 5 days, followed by a second exposure of another 5 days.

	Drug/Inhibitor	First exposure	Second exposure
**Control**	PAT	PAT	PAT
**Combination**	AMB	PAT + AMB	PAT + AMB
**Serial**		PAT	PAT + AMB
**Combination**	MIL	PAT + MIL	PAT + MIL
**Serial**		PAT	PAT + MIL
**Combination**	FCCP	PAT + FCCP	PAT + FCCP
**Serial**		PAT	PAT + FCCP
**Combination**	BOR	PAT + BOR	PAT + BOR
**Serial**		PAT	PAT + BOR
**Combination**	PARO	PAT + PARO	PAT + PARO
**Serial**		PAT	PAT + PARO

Supplementary Table 8. Experimental design for the extended study of BOR tolerance. First exposure is made for 5 days, followed by a second exposure of another 5 days, PAT is used at a normally lethal dose; BOR is used at 1x, 2x and 5 x IC50.

	Drug/Inhibitor	First exposure	Second exposure
**Control**	PAT	PAT	PAT
**Combination**	BOR	PAT + BOR	PAT + BOR
**Serial**		PAT	PAT + BOR
**Combination**	2 x BOR	PAT + BOR	PAT + BOR
**Serial**		PAT	PAT + BOR
**Combination**	5 x BOR	PAT + BOR	PAT + BOR
**Serial**		PAT	PAT + BOR

Data Sets

The data sets 1-7 are available as excel files in the submission package

Supplementary Data 1. Sequencing statistics (RNAseq). For each sample, Total_reads represents the total number of sequenced fragments (paired-end read pairs). Fragment classification is based on htseq-count output. no_feature_reads indicates fragments that were successfully aligned to the reference genome but did not overlap any annotated exon. Ambiguous_Reads corresponds to fragments overlapping exons from more than one gene and therefore not uniquely assignable. Low_Quality_Reads includes fragments discarded due to low mapping quality. Not_Aligned_Reads represents fragments for which no usable alignment was available after filtering. percentage_mapped indicates the proportion of fragments with at least one mapped read. Mapped_to_features denotes the number of fragments uniquely assigned to annotated exonic features (gene counts), and percentage_mapped_to_feature represents the proportion of total fragments successfully assigned to features.

Supplementary Data 2. V1 vs V2 gene annotation. First and second column contains the gene identifier in the version 2 and version 1 of the *L. donovani* BPK282 reference genome. V1 is the version available in TriTryp DB; V2 is the reference genome developed in our lab and published [1]

Supplementary Data 3. Raw RNA-seq read counts per sample prior to normalization. Paired-end reads were aligned using BWA, and gene-level counts were generated with htseq-count based on GFF annotations.

Supplementary Data 4. Changes in gene expression in different experimental conditions: Excel file containing differential expression results generated using DESeq2 for all analyzed conditions. The "source" sheet includes the complete filtered DESeq2 output, with gene identifiers (geneID), log2 fold changes (FC), false discovery rates (FDR), and normalized expression values (log-transformed RPKM) for each condition (noPAT, PAT D2, PostPAT for both strain BPK026 and PBK275). For each condition, additional sheets report subsets of differentially expressed genes based on the following thresholds:

absolute log2 fold change ≥ 1 and FDR ≤ 0.05. Sheets labeled "condition – up" contain significantly upregulated genes (FC ≥ 1), "condition – down" contain significantly downregulated genes (FC ≤ −1), and "condition – all" contain all significant genes irrespective of direction. Sheets labeled "common up", "common down", and "common all", which list genes consistently differentially expressed across conditions in both strains.

Supplementary Data 5. KEGG pathway enrichment per condition based on differentially expressed genes, as derived from Data set 4. Enrichment was tested using a hypergeometric model with size and detection cutoffs applied; only significantly enriched pathways (adjusted $p < 0.05$) are shown. One worksheet per condition.

Supplementary Data 6. Sequencing statistics (whole genome sequencing). For each sample, the total number of raw reads, mapped reads, and the percentage of mapped reads are shown. QC-processed mapped reads represent alignments retained after sequential filtering for mapping quality > 30 (MAPQ > 30), removal of duplicate reads, and properly paired reads. Percentages are calculated relative to the total number of raw reads per sample.

Supplementary Data 7. Sheet A. SNPs present in three replicates of the P1 and P3/P4 post SbIII strains [PAT] and not detected in corresponding no-drugged controls (no-PAT); 0, as reference genome; 1, heterozygous SNP; 2, homozygous SNP. Sheet B. The analysis was repeated with lower stringency, considering SNPs common to at least two replicates (and not detected in controls).

Source data for figures are stored in Zenodo; https://doi.org/10.5281/zenodo.18663711

SUPPLEMENTARY FIGURES

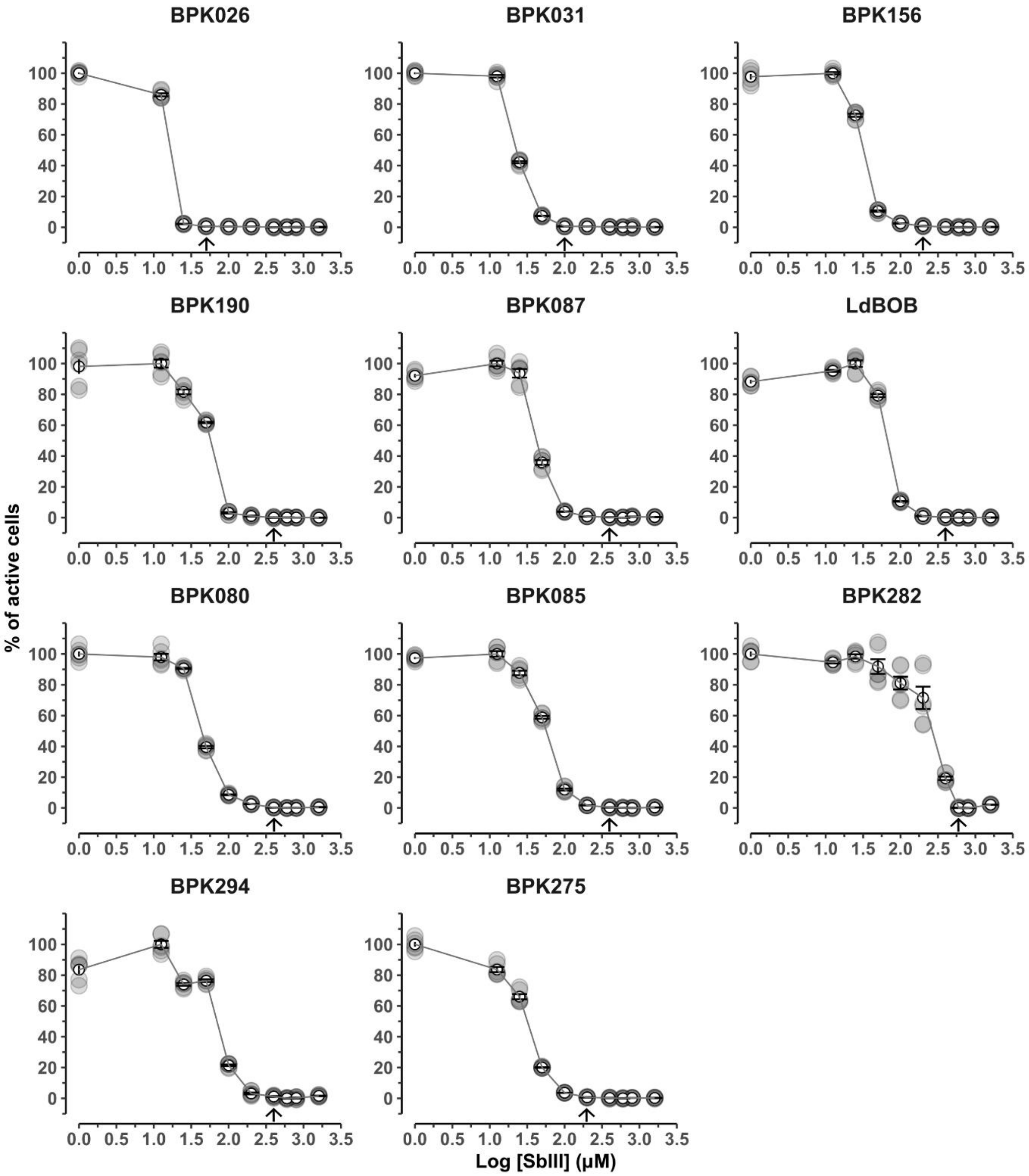

Supplementary Figure 1. Definition of the normally lethal dose of antimonials for each strain. Percentage of active cells in function of the PAT concentration measured to define the lowest PAT concentration (arrow) at which no metabolic activity could be detected (see also Supp. Table 1), further called normally lethal dose. Results are represented as the mean of 3 biological replicates ± SEM, with the corresponding individual data points displayed as semi-transparent symbols for each strain.

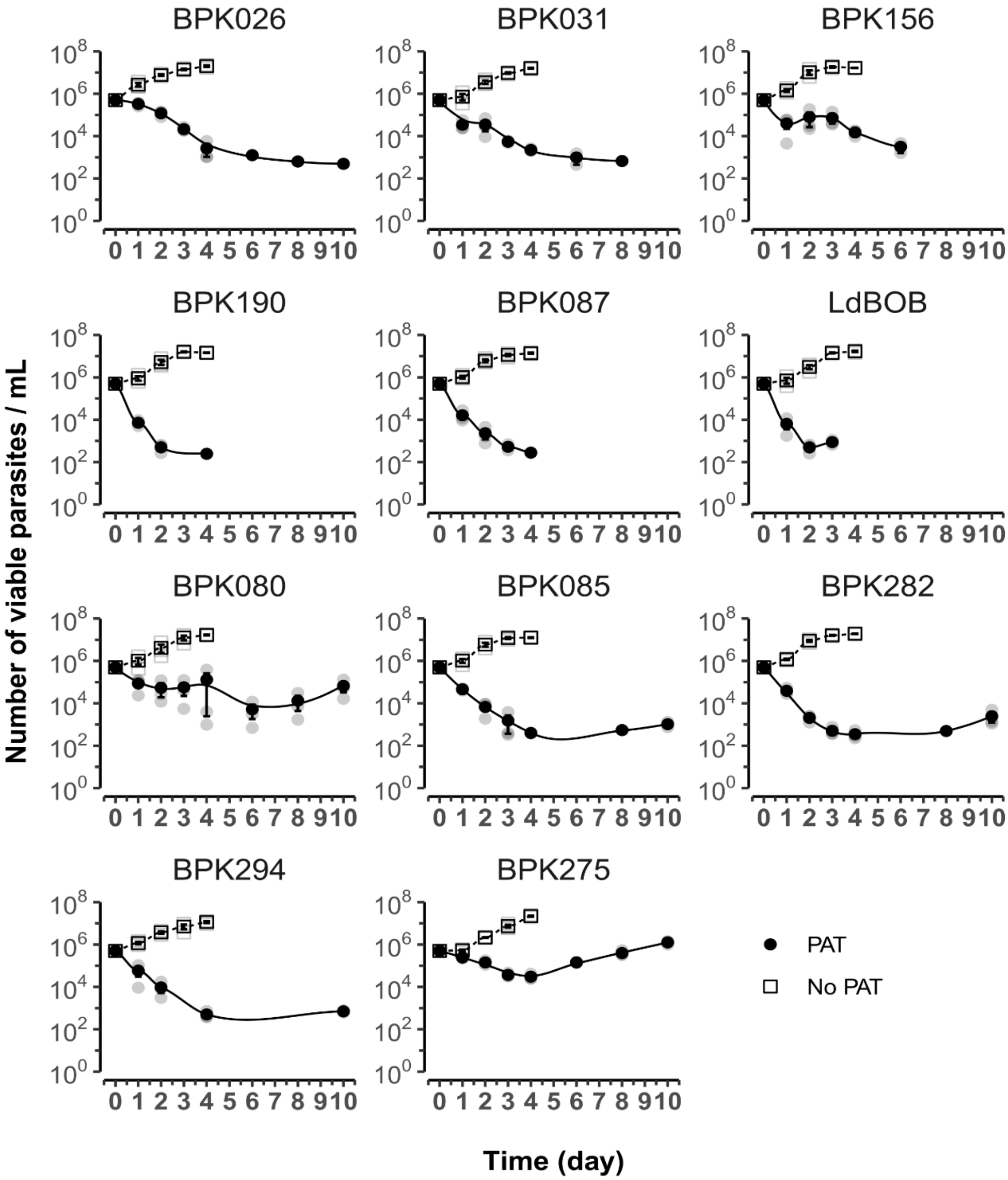

Supplementary Figure 2. Growth curve of *L. donovani* strains in normally lethal PAT doses. The number of viable promastigotes per mL during 10 days of PAT exposure (PAT), and a control of no drug exposure (No PAT) is included. The graph illustrates the mean ± SEM of three biological replicates for daily measurement, with the corresponding individual data points displayed as semi-transparent symbols.

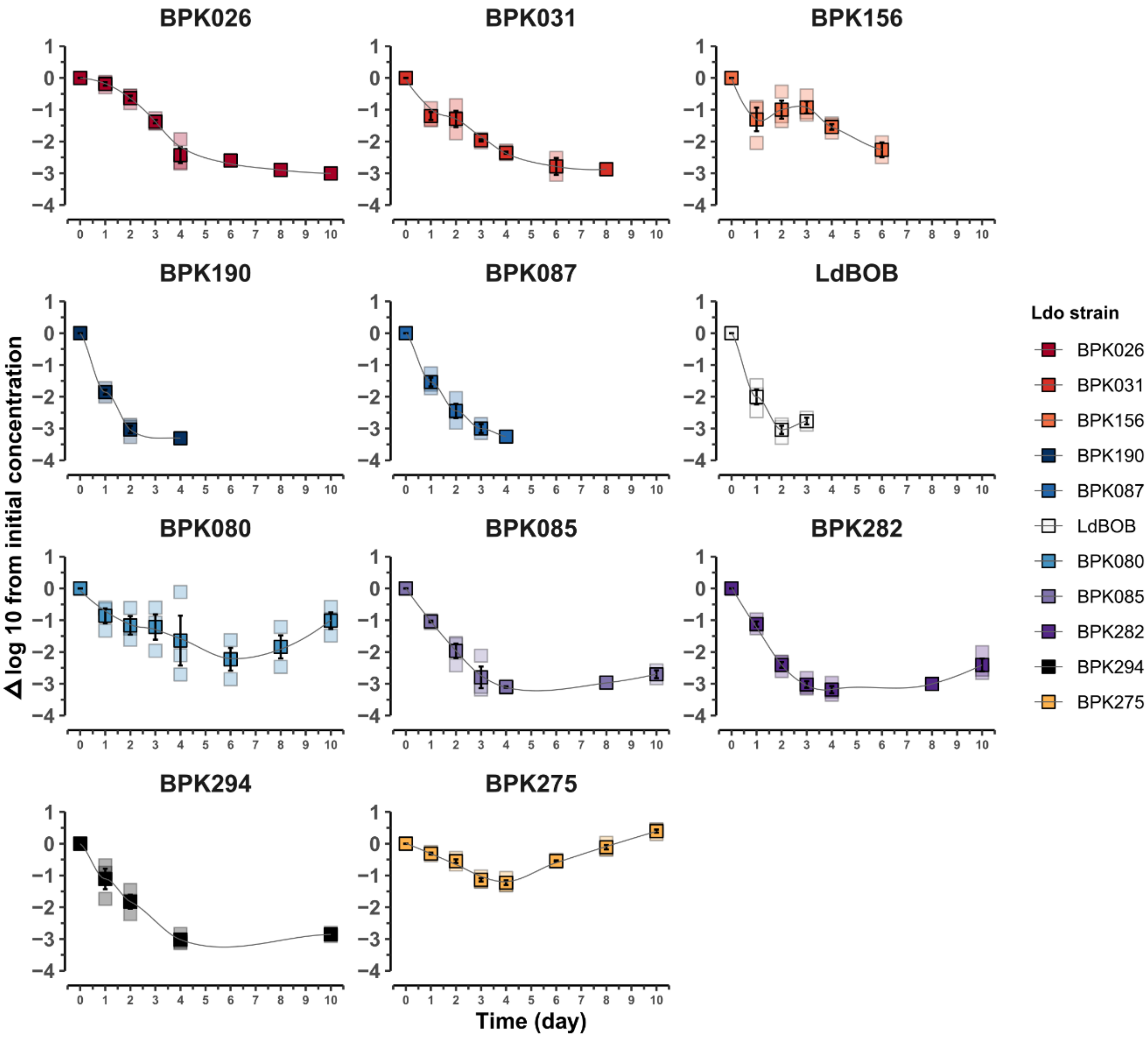

Supplementary Figure 3. Survival of *L. donovani* strains under PAT pressure. The number of viable promastigotes was measured by flow cytometry under 10-days exposure at strain-specific normally lethal doses of PAT. The values were calculated as the reduction in parasite concentration relative to the initial inoculum and are presented as the mean ± SEM of three biological replicates, with the corresponding individual data points shown as semi-transparent symbols for each strain.

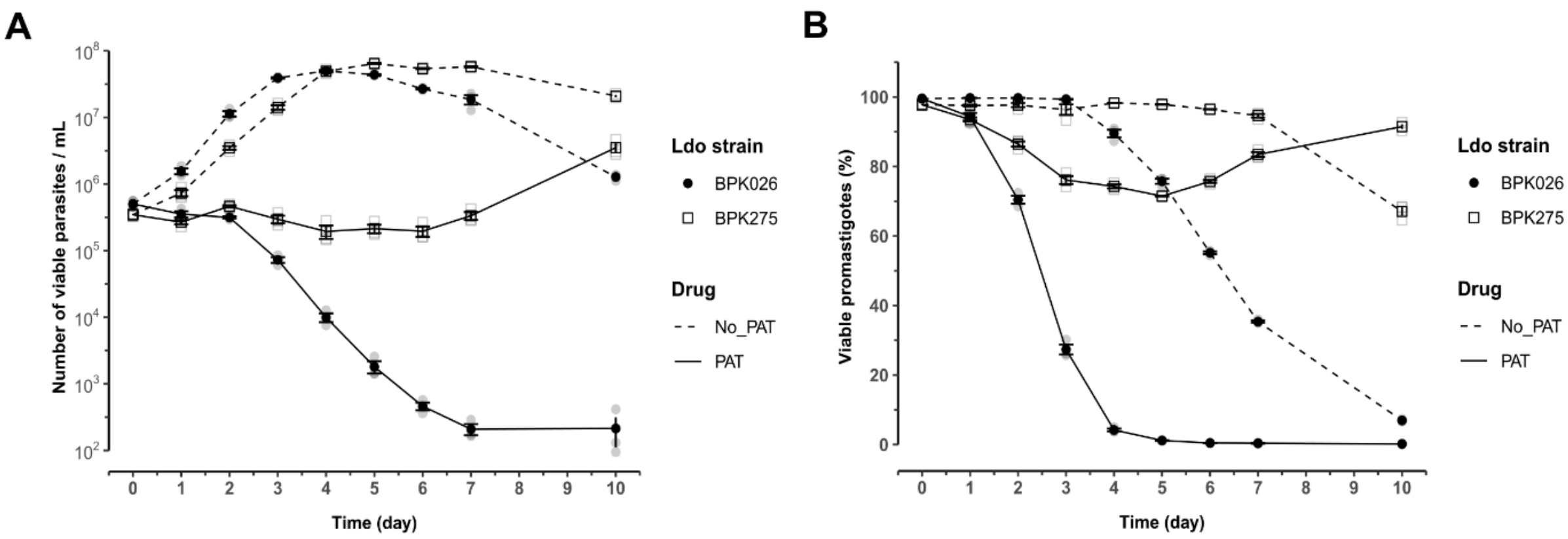

Supplementary Figure 4. Growth and viability of promastigotes in normally lethal dose of PAT. (A) Growth curve of promastigotes and, (B) Percentage of cell viability along 10 days of PAT exposure (PAT, solid line). A control of no drug exposure (No PAT, dashed line) is included. The data was acquired by flow cytometry for BPK026 (circle symbol) and BPK275 (square symbol). The graph illustrates the mean ± SEM of three biological replicates for daily measurement, with the corresponding individual data points displayed as semi-transparent symbols.

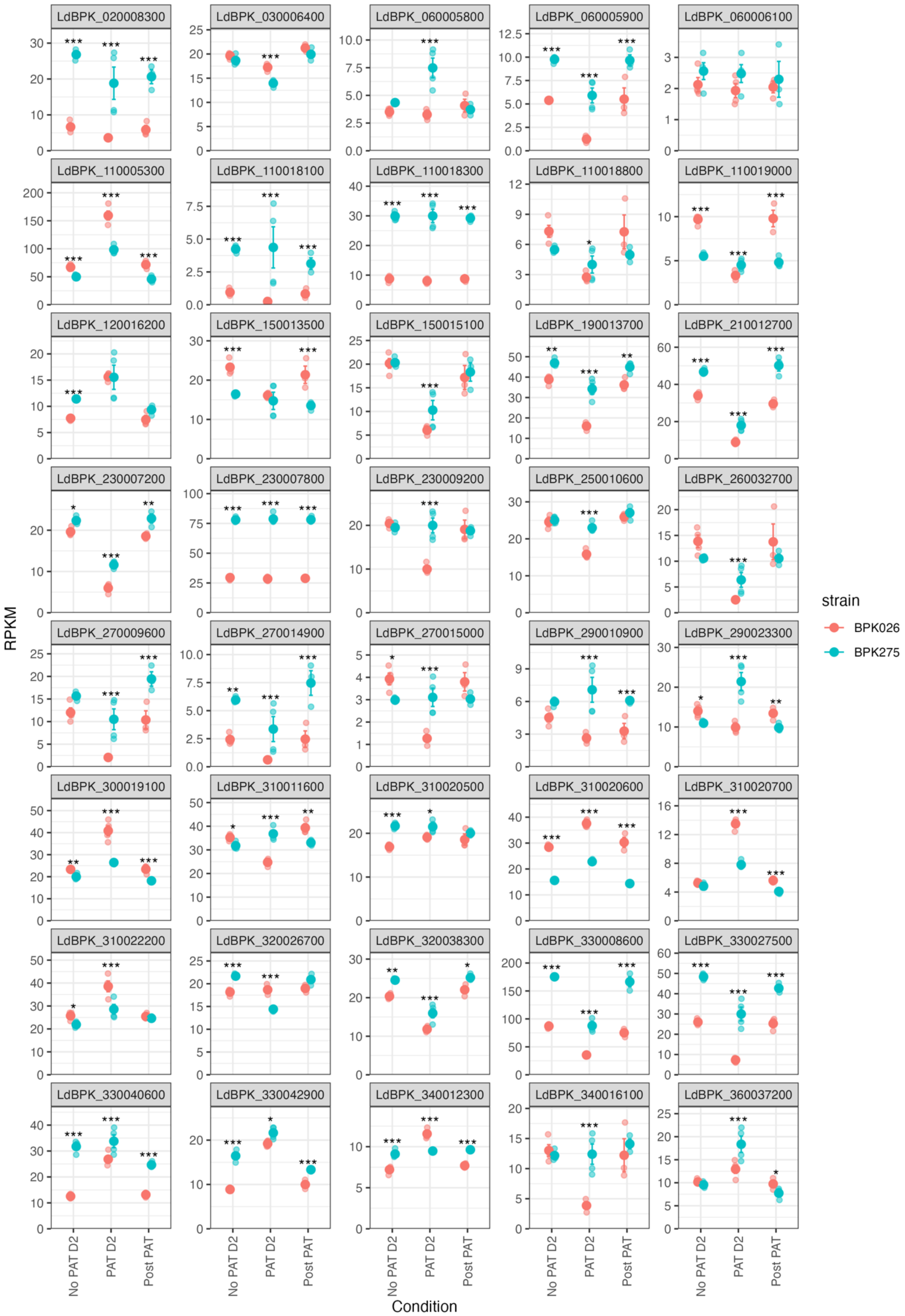
LdBPK_020008300
LdBPK_030006400
LdBPK_060005800
LdBPK_060005900
LdBPK_060006100
LdBPK_110005300
LdBPK_110018100
LdBPK_110018300
LdBPK_110018800
LdBPK_110019000
LdBPK_120016200
LdBPK_150013500
LdBPK_150015100
LdBPK_190013700
LdBPK_210012700
LdBPK_230007200
LdBPK_230007800
LdBPK_230009200
LdBPK_250010600
LdBPK_260032700
LdBPK_270009600
LdBPK_270014900
LdBPK_270015000
LdBPK_290010900
LdBPK_290023300
LdBPK_300019100
LdBPK_310011600
LdBPK_310020500
LdBPK_310020600
LdBPK_310020700
LdBPK_310022200
LdBPK_320026700
LdBPK_320038300
LdBPK_330008600
LdBPK_330027500
LdBPK_330040600
LdBPK_330042900
LdBPK_340012300
LdBPK_340016100
LdBPK_360037200
RPKM
No PAT D2
PAT D2
Post PAT
Condition
strain
BPK026
BPK275

Supplementary Figure 5. Expression of ABC transporters. Dot plots show RPKM (Reads per Kilobase Million) expression levels for 40 ABC transporter genes across three conditions: No PAT D2, PAT D2, and Post PAT. Each dot represents an individual biological replicate. Colors indicate strain (BPK026, red; BPK275, green). Larger points indicate group means, with vertical error bars representing the standard error of the mean (SEM). Within each gene, statistical significance between the two strains for a given condition is indicated by asterisks (* $p < 0.05$; ** $p < 0.01$; *** $p < 0.001$). The 14 ABC transporters showing higher transcript abundance in BPK275 both with and without drug exposure are: LdBPK-020008300, -060005900, -110018100, -110018300, -190013700, -210012700, -230007200, -230007800, (MRPA), -270014900, -320038300, -330008600, -330027500, -330040600 and -330042900. The 11 ABC transporters that showed higher abundance in BPK275 as compared to BPK026 specifically during the quiescence period (D2) are: LdBPK-060005800, -110018800, -110019000, -150015100, -230009200, -250010600, -260032700, -270015000, -290023300, -310011600, -340016100.

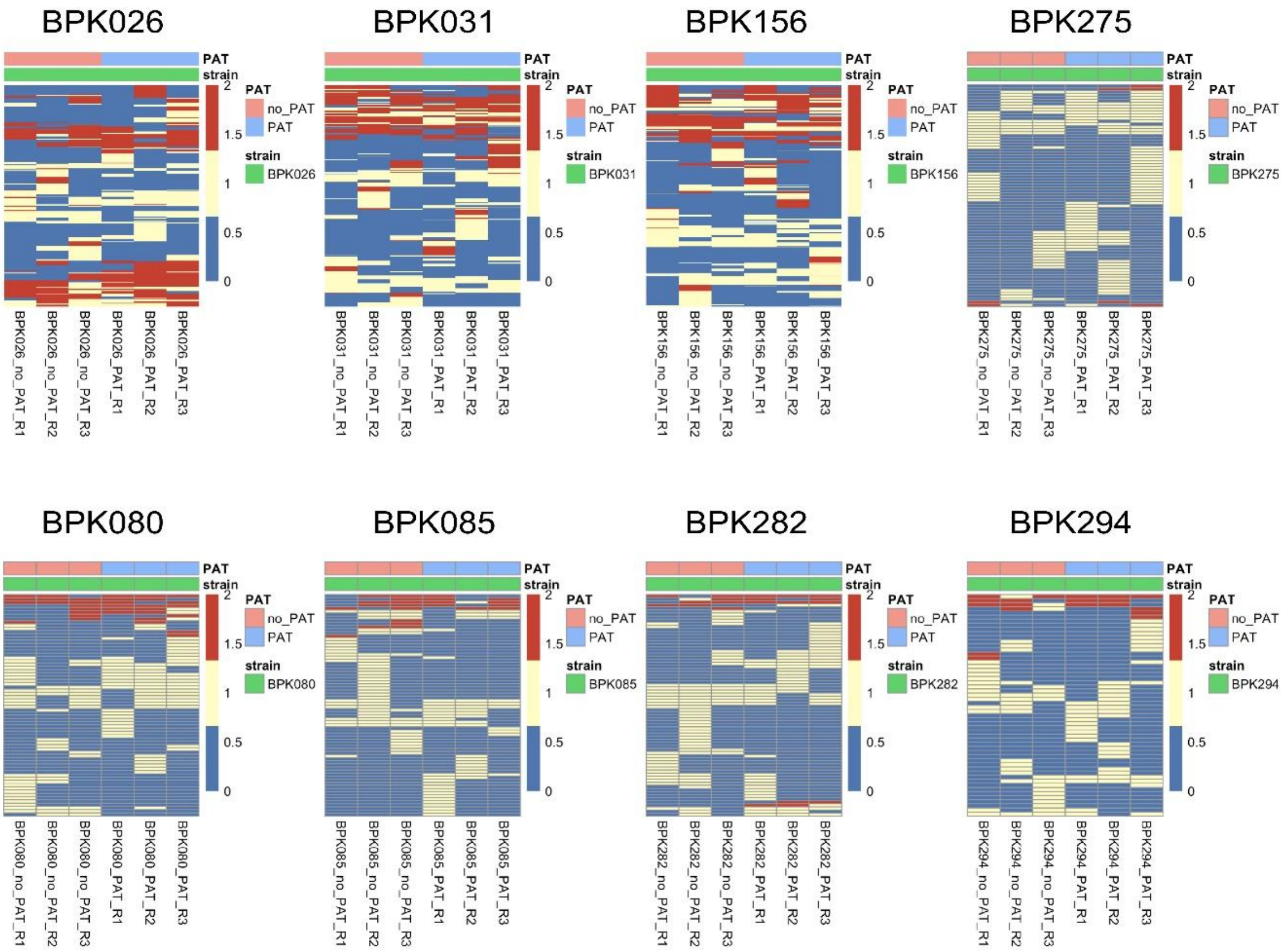

Supplementary Figure 6. Heatmap illustrating the distribution of single nucleotide polymorphisms (SNPs) per individual strain. Only SNPs with an impact – determined using SnpEff – on the coding region are retained. SNPs that exhibited no variation across the six replicates per strain were filtered out. The color scheme categorizes SNPs as follows: blue represents the absence of a SNPs, yellow indicates heterozygous SNPs, and red denotes homozygous SNPs. Hierarchical clustering of samples (columns) and SNPs (rows) was performed using the complete linkage method with Euclidean distance. A color-coded bar above the heatmap indicates whether each strain was exposed to PAT, alongside the strain names. The graphs represents three biological replicates (R1, R2, R3) after removing PAT exposure, along with their corresponding controls without exposure (no_PAT).

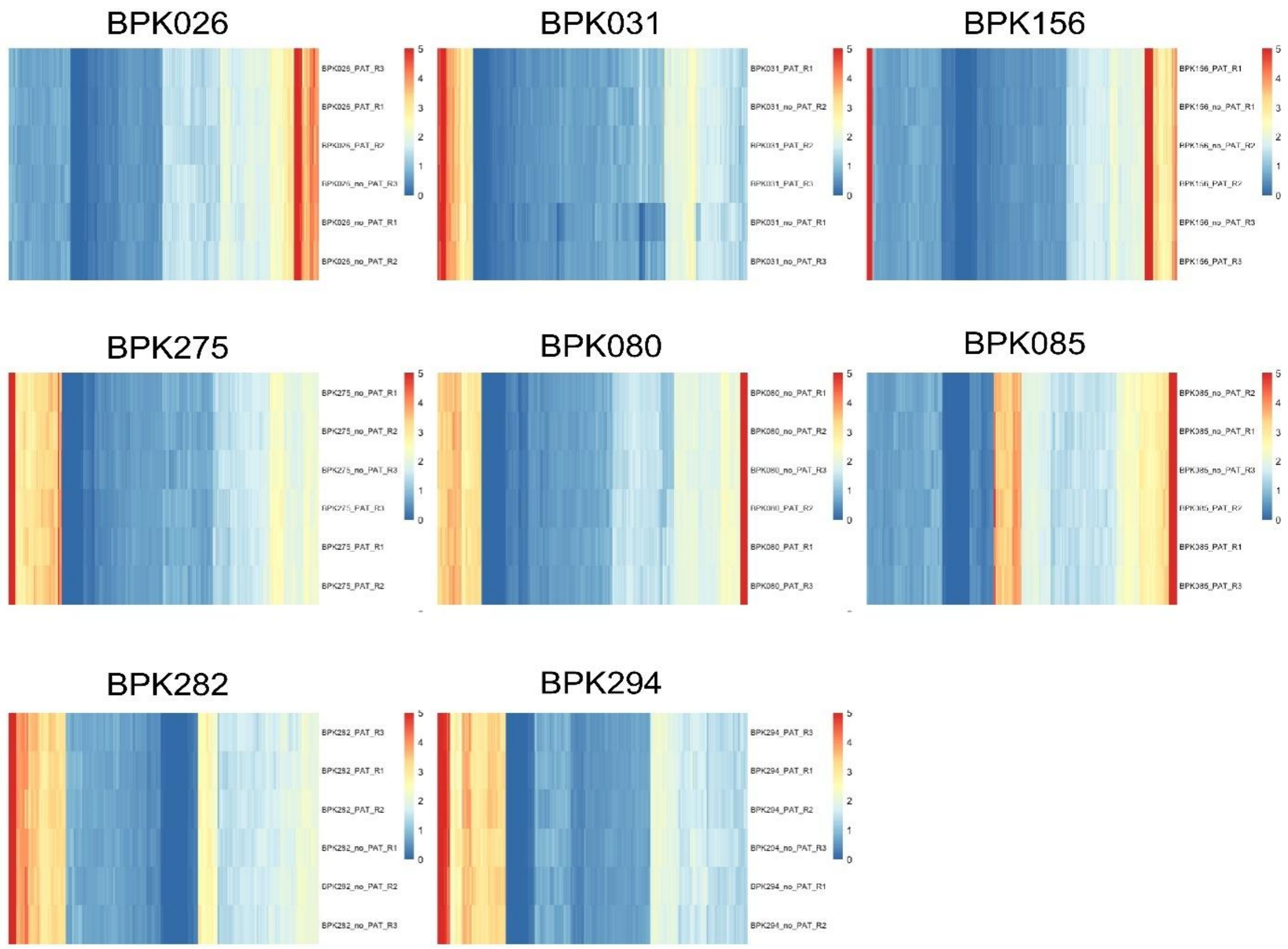

Supplementary Figure 7. Heatmap for each strain separately displaying CNV values for genes with a 50% increase or decrease in CNV (no statistical significance required).

A.

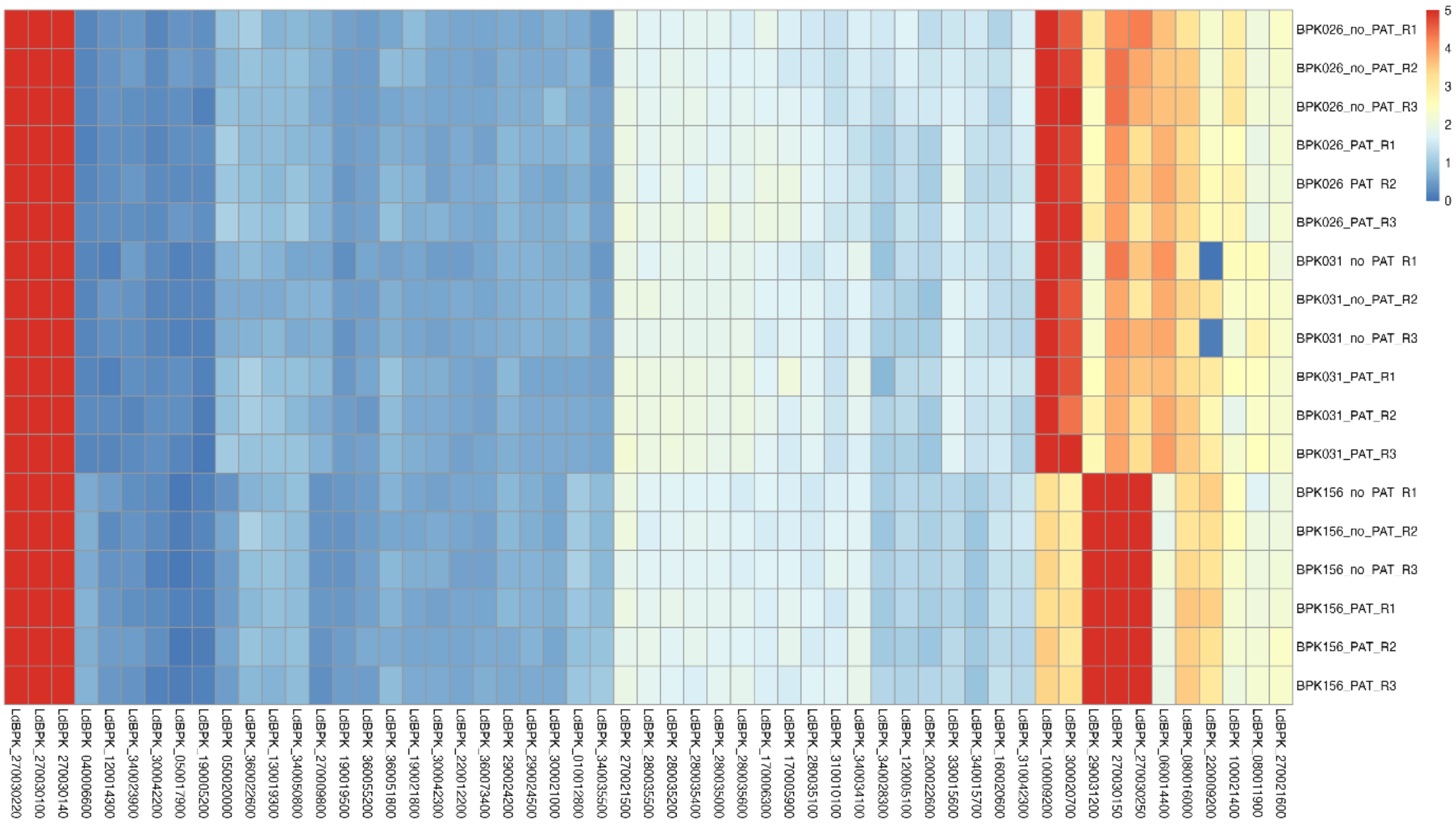

B.

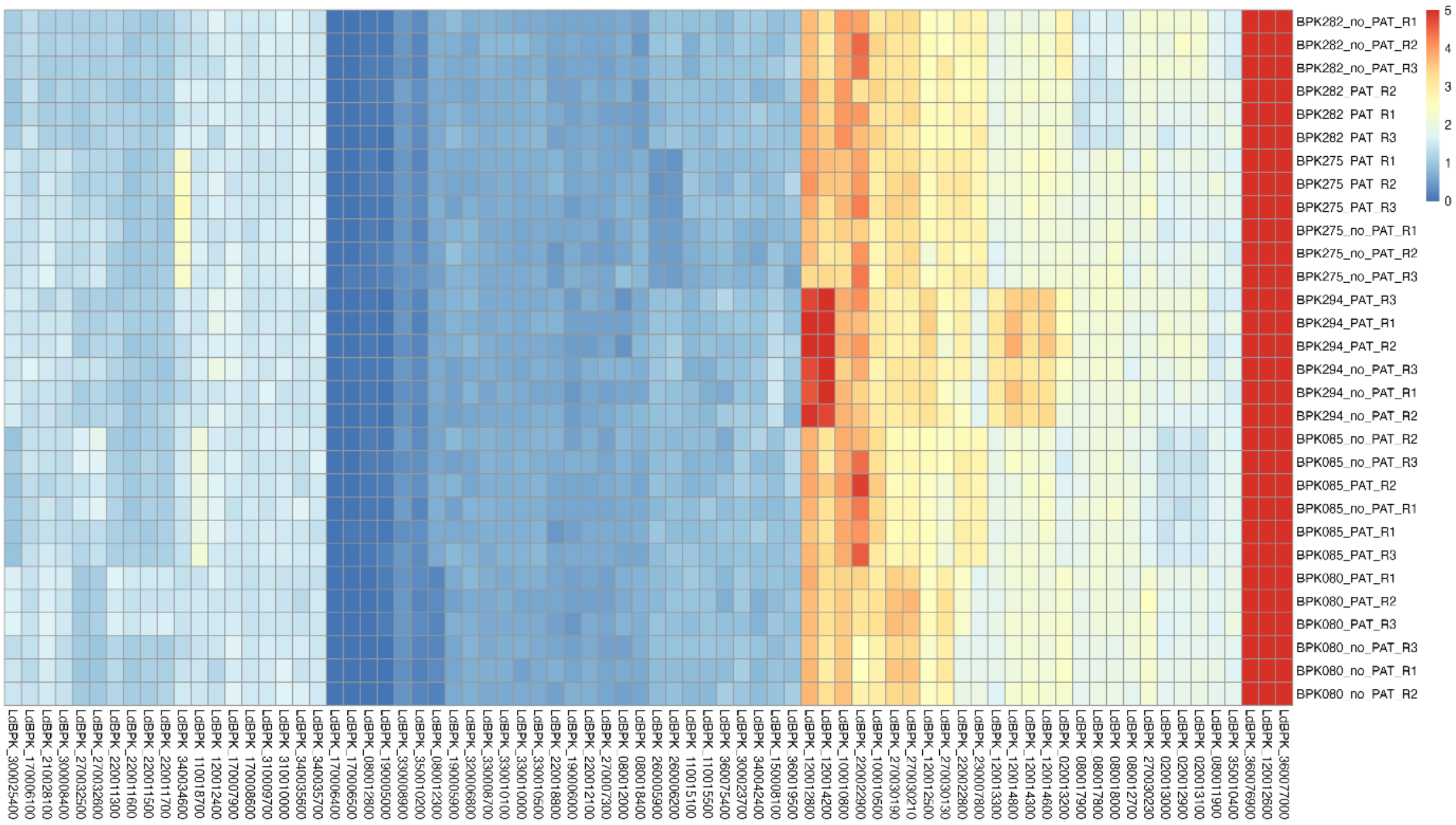

Supplementary Figure 8. **Heatmap of the** (**A**) Profile 1 and (**B**) Profiles 3/4 strains, displaying CNV values for genes with a statistically significant 50% increase or decrease in CNV. Statistical significance was determined using a Student's t-test to compare CNV values between PAT-exposed (PAT), and non-

exposed replicates (No_PAT) for each strain. Three biological replicates were included (R1,R2,R3) for each strain and drug exposure.

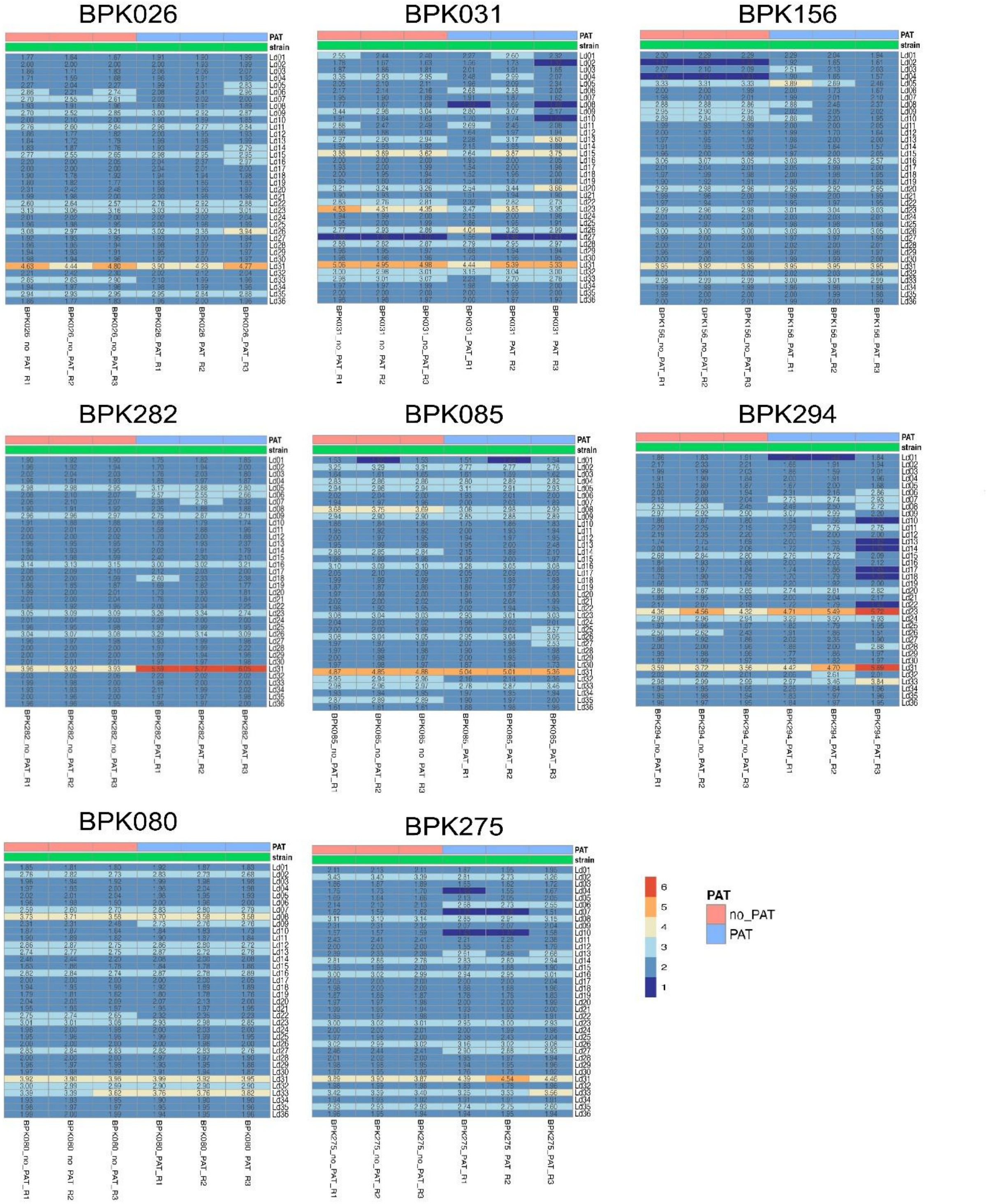

Supplementary Figure 9. Heatmap showing the somy value per replicate, chromosome, and strain. A color-coded bar above the heatmap indicates whether each strain was exposed to PAT. Three biological replicates are indicated as R1, R2, and R3.

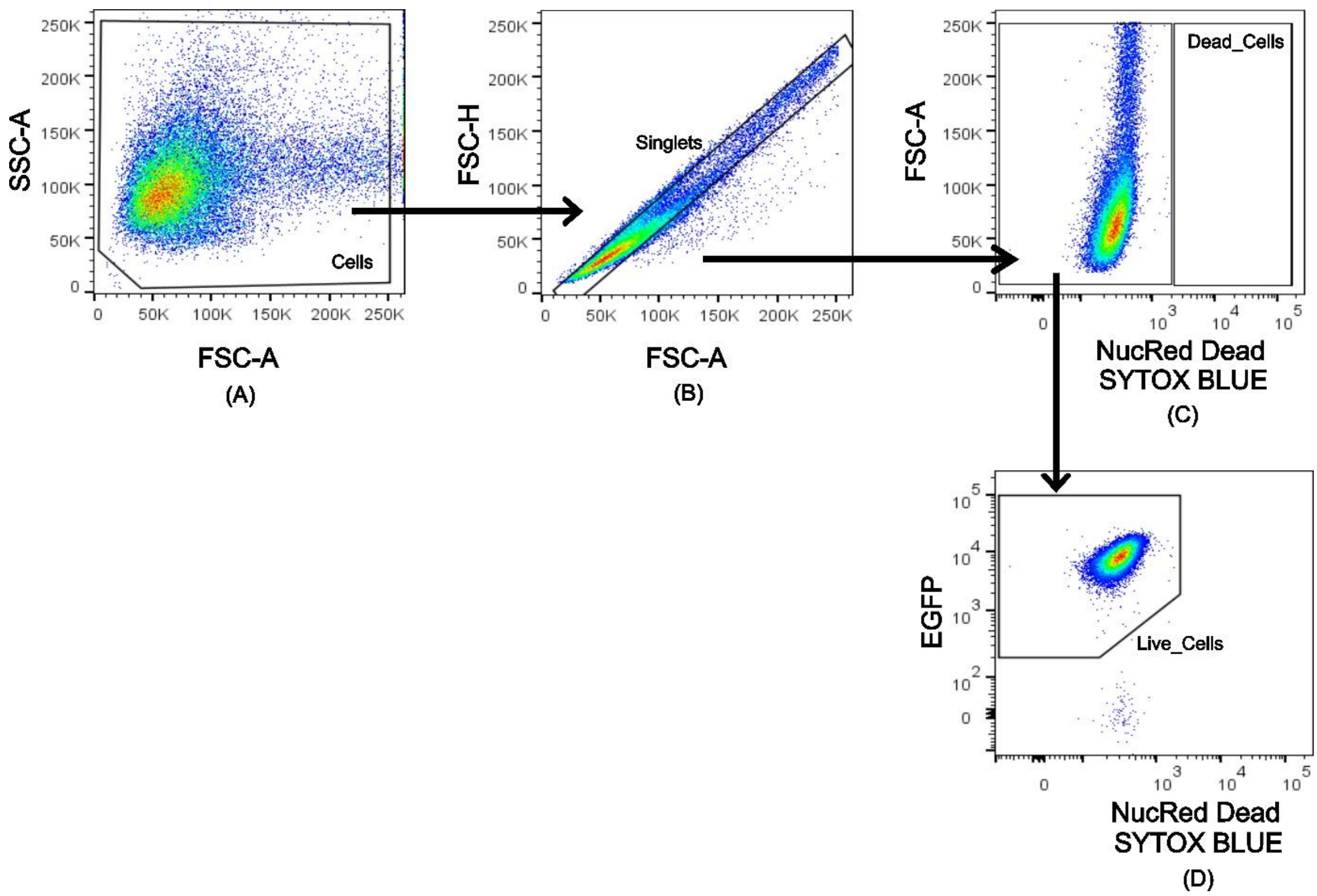

Supplementary Figure 10. Gating strategy for the analysis of *Leishmania* promastigotes. (**A**) Total cells were identified based on forward scatter (FSC) area and side scatter (SSC) area. (**B**) Then, singlets were selected by analyzing FSC height over FSC area. (**C**) The negative cells for NucRed Dead or SYTOX BLUE (viability markers) were chosen to exclude dead cells. (**D**) The population of cells positive for EGFP and negative for NucRed Dead or SYTOX BLUE was determined as live cells for further analysis.

SUPPLEMENTARY TEXT

Transcriptional analyses

To obtain comprehensive overview of transcriptional adaptations during adoption of quiescence we performed differential expression analysis using DESeq2 package for several pairwise comparisons: i) to identify transcripts differentially expressed in quiescent cells by comparing PAT D2 versus No PAT; first specific for BPK026 and BPK275, and then also common for both strains ii) to address whether recovered parasites (Post-PAT) were transcriptionally comparable to no PAT parasites, as would be expected from cells transitioning through a short period of quiescence, iii) to identify overall molecular differences between BPK026 and BPK275, by comparing these two strains with particular focus on PAT D2 timepoint. The summary of differentially expressed genes for all these comparisons can be found in Supp. Table 4 and Fig.3C, and the full lists of differentially expressed genes in all comparisons can be found in Data sets 2-4.

Genomic analyses

Because of ISC1 strains/P1 parasites having much higher number of heterozygous SNPs in comparison to other strains [2], we carried out the analysis of P1 and P3/4 parasites separately; but we searched SNPs present in three replicates of post SbIII and not detected in non-drugged controls (Post-PAT). In both P1 and P3/4 lines, we observed 56 SNPs but did not observe consistent (*i.e.* present in all three replicates) SNP/indel changes (Supp. Fig. 6), including in intergenic regions of *AQP1* and *MRPA*. The analysis was repeated with lower stringency, considering SNPs common to at least two replicates (and not detected in controls). This revealed more hits (321), but we found no variants (linked to the same locus), which were specific to P3/P4 parasites, and as such could underlie the observed hyper-resistance (see supplement and Data set 7). With respect to CNVs there were no consistent changes in the depth of individual coding sequences (Supp. Fig. 7 & 8), including the *MRPA* genes (Data set 7) when No- and Post-PAT parasites were compared. However, when we analyzed somy changes in Post-

PAT parasites as compared to non-exposed lines, we observed a general decrease of somy of several chromosomes in both P1 and P3/4 lines (Supp. Fig. 9 and Fig. 4). Focusing on the somy changes that we consider biologically significant (S values difference of at least 0.5, p-value$\leq$0.05), we detected: i) decrease in at least one chromosome in all P1 parasites, namely chr 33 and chr 07 in BPK026, chr 23 in BPK031 and chr 09 in BPK156. In addition, five chromosomes, chr 02, chr 08, chr 14, chr 32 and chr 35 were decreased in BPK085, and chr 26 in BPK294 ii) no significant increases in chromosome copy number were found in P1 parasites, while we observed a significant increase in copy number of chr 6 and chr 31 in BPK282 and BPK275. In addition, an increase (albeit not reaching statistical significance) in chr 31 was found in BPK294, where chr 07 was also increased. No significant aneuploidy changes were detected in BPK080.

Models for future studies

The 11 *L. donovani* lines studied here exhibited a range of quiescence and persister phenotypes, which may be exploited further to probe the molecular mechanism that control the transition to, maintenance and exit from quiescent state. For example, BPK087 did not exhibit reduction of rEGFP expression in the very early timepoints of exposure, resulting in rapid killing. This suggests that this line was not able to enter quiescence in our experimental conditions. It would be interesting to see whether this remains the same if different doses of PAT are applied, or if other routes to quiescence e.g. purine starvation[3] were applied. If BPK087 proves to be unable to enter into quiescence in response to PAT pressure, it could serve as a useful model to uncover signalling that leads to quiescence entry. At the same time, several lines characterized by profile 3 exhibited the ability to exit quiescence even while still under drug pressure and could be employed to uncover the mechanisms controlling the maintenance and promoting the exit from the quiescent state.

BIBLIOGRAPHY

1. Dumetz, F. *et al.* Modulation of aneuploidy in leishmania donovani during adaptation to different in vitro and in vivo environments and its impact on gene expression. *mBio* **8**, e00599-17 (2017).
2. Imamura, H. *et al.* Evolutionary genomics of epidemic visceral leishmaniasis in the Indian subcontinent. *Elife* **5**, (2016).
3. Martin, J. L. *et al.* Metabolic Reprogramming during Purine Stress in the Protozoan Pathogen Leishmania donovani. *PLoS Pathog.* **10**, e1003938 (2014).
