# Supplementary material for: Multi-drug tolerance in Leishmania persister-like cells
Source: Commun Biol. 2026 Jun 12;9:1263. doi: 10.1038/s42003-026-10456-w (PMC13616925; doi:10.1038/s42003-026-10456-w)
Supplement: Supplementary file 4 [file 42003_2026_10456_MOESM2_ESM.docx]

**Description of Additional Supplementary File**

File name: Supplementary Data 1
Description: Sequencing statistics (RNAseq).

File name: Supplementary Data 2
Description: V1 vs V2 gene annotation

File name: Supplementary Data 3
Description: Raw RNA-seq read counts per sample prior to normalization

File name: Supplementary Data 4
Description: Changes in gene expression in different experimental conditions

File name: Supplementary Data 5
Description: KEGG pathway enrichment

File name: Supplementary Data 6
Description: Sequencing statistics (whole genome sequencing).

File name: Supplementary Data 7
Description: SNPs present in post SbIII strains [PAT] and not in no-drugged controls [noPAT]
